# Fragment Screening at Adenosine-A_3_ Receptors in Living Cells Using a Fluorescence-Based Binding Assay

**DOI:** 10.1016/j.chembiol.2012.07.014

**Published:** 2012-09-21

**Authors:** Leigh A. Stoddart, Andrea J. Vernall, Jessica L. Denman, Stephen J. Briddon, Barrie Kellam, Stephen J. Hill

**Affiliations:** 1 Institute of Cell Signalling, School of Biomedical Science, Queen's Medical Centre, University of Nottingham, Nottingham NG7 2UH, UK; 2 School of Pharmacy, Centre for Biomolecular Sciences, University of Nottingham, Nottingham NG7 2RD, UK

## Abstract

G protein-coupled receptors (GPCRs) comprise the largest family of transmembrane proteins. For GPCR drug discovery, it is important that ligand affinity is determined in the correct cellular environment and preferably using an unmodified receptor. We developed a live cell high-content screening assay that uses a fluorescent antagonist, CA200645, to determine binding affinity constants of competing ligands at human adenosine-A_1_ and -A_3_ receptors. This method was validated as a tool to screen a library of low molecular weight fragments, and identified a hit with submicromolar binding affinity (K_D_). This fragment was structurally unrelated to substructures of known adenosine receptor antagonists and was optimized to show selectivity for the adenosine-A_3_ receptor. This technology represents a significant advance that will allow the determination of ligand and fragment affinities at receptors in their native membrane environment.

## Graphical abstract

**Graphical Abstract:**

**Figure:**
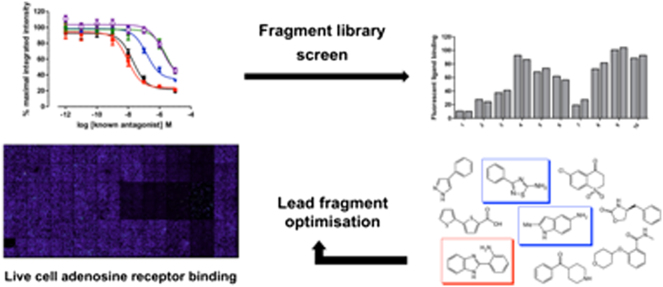

**Highlights:**

► Fluorescence-based competition binding assay for adenosine-A_1_ and -A_3_ receptors ► Fragment screen using receptors in the native membrane environment in living cells ► Lead compound identification and optimization from a commercial fragment library

## Teaser

G protein-coupled receptors are important drug targets, and initiatives that measure ligand affinity in the native cellular environment are required. Here, Stoddart et al. report a live cell, high content, fluorescence-based assay to measure ligand affinity at the human adenosine-A1 and -A3 receptors.

## Introduction

G protein-coupled receptors (GPCRs) comprise the largest family of transmembrane proteins and represent major targets for drug discovery, with over 40% of currently marketed drugs acting at these cell surface receptors. Considerable advances in our knowledge of GPCR structure have been recently achieved from X-ray crystallography (Cherezov et al., 2007; Chien et al., 2010; Hanson et al., 2012; Jaakola et al., 2008; Rasmussen et al., 2011; Shimamura et al., 2011). This has led to insights into the conformational changes that result during receptor activation (Chung et al., 2011; Rasmussen et al., 2011) and has provided opportunities for virtual screening of molecular libraries and fragment-like ligands (de Graaf et al., 2011; Kolb et al., 2009). In addition, availability of highly purified detergent-solubilized receptor protein has enabled fragment screening using biophysical approaches such as surface plasmon resonance and nuclear magnetic resonance (Congreve et al., 2011). However, the act of detergent solubilization disrupts the local environment in which these membrane proteins normally reside and removes many of the ancillary proteins that can provide allosteric influences on ligand-receptor interactions (Kenakin, 2012).

It is now acknowledged that GPCRs can adopt multiple active conformations as a consequence of protein-protein interactions that can lead to the activation or attenuation of different signaling pathways (Kenakin and Miller, 2010; Swaminath et al., 2004). Furthermore, different agonists appear able to bias signaling in favor of a particular downstream pathway, including those that do not involve heterotrimeric G proteins (Azzi et al., 2003; Baker et al., 2003; Whalen et al., 2011). It is also clear that the binding affinity of antagonists can vary depending on the signaling pathway and agonist that is being studied (Baker and Hill, 2007). These data suggest that intracellular signaling proteins can elicit marked allosteric influences on the binding of both agonists and antagonists to a particular GPCR (Kenakin and Miller, 2010; Kenakin, 2012; Williams and Hill, 2009) and as a consequence the cellular context in which binding affinities are measured will have a major impact on drug screening strategies. It is therefore imperative to derive methods for the measurement of ligand-binding affinity in living cells, where the integrity of the local membrane environment and receptor is maintained under physiologic conditions.

Fluorescence-based assays have the sensitivity and resolution to monitor ligand-binding in single living cells, and high-quality fluorescent ligands for GPCRs are now becoming available (Daly et al., 2010; May et al., 2010; Middleton and Kellam, 2005). The adenosine-A_3_ receptor (A_3_AR) belongs to a family of four GPCRs (A_1_, A_2A_, A_2B_, and A_3_) (Fredholm et al., 2011) that respond to adenosine and are attractive drug targets for a number of pathophysiologic conditions including cancer, ischemia, cardiovascular disease, and inflammation. We have shown that fluorescent BODIPY630/650 (BY630) labeled agonists can be used to monitor the kinetics of ligand-binding to unmodified human adenosine-A_1_ (A_1_AR) and A_3_AR receptors in real time at the single cell level by taking advantage of the marked increase in quantum yield of the BODIPY fluorophore in the local membrane environment of the receptor that occurs as the ligand binds (May et al., 2010, 2011). We developed a competition binding assay using a novel fluorescent antagonist and a high-content screening system for the automated capture and analysis of images. We show that measuring total image intensity allowed accurate affinity values of antagonists at the A_1_AR and A_3_AR to be determined. Furthermore, we demonstrate that the assay can detect weakly binding low molecular weight compounds.

The ability of the assay to detect low-affinity ligand-binding molecules suggested that the assay may be amenable for fragment screening approaches. Fragment-based drug discovery uses a chemical library containing only small, low molecular weight compounds and is a powerful and widely used tool in drug discovery because it explores chemical space more efficiently. A fragment screen often results in lead compounds with a low molecular weight and complexity; desirable characteristics for a drug candidate lead (Hopkins et al., 2004). Biophysical methods such as X-ray crystallography, nuclear magnetic resonance spectroscopy, surface plasma resonance, and mass spectrometry are most commonly used for fragment screening (Bartoli et al., 2006), and have been successfully used to generate novel lead compounds against cytosolic proteins such as kinases and proteases (Whittaker et al., 2010). While structural information is now becoming available on GPCRs (Cherezov et al., 2007; Chien et al., 2010; Hanson et al., 2012; Jaakola et al., 2008; Rasmussen et al., 2011; Shimamura et al., 2011), the importance of the membrane environment for their functional integrity and stability has made them less amenable to analysis with biophysical techniques. Some success has been achieved using highly purified detergent-solubilized receptor protein that has been thermostabilized with genetic mutations (Congreve et al., 2011). However, a major concern is that this removes the GPCR from the intracellular signaling proteins that may allosterically modify ligand-binding behavior (Chung et al., 2011; Kenakin, 2012; Steyaert and Kobilka, 2011; Williams and Hill, 2009). In this study, we show that measurement of the binding of a fluorescent ligand to both A_1_AR and A_3_AR in living cells can be used to screen a fragment library and identify molecules with micromolar affinity, which can discriminate between these two closely related receptor subtypes.

## Results

### A Fluorescent Xanthine Amine Congener, CA200645, as a Fluorescent A_3_AR Antagonist

**Figure 1:**
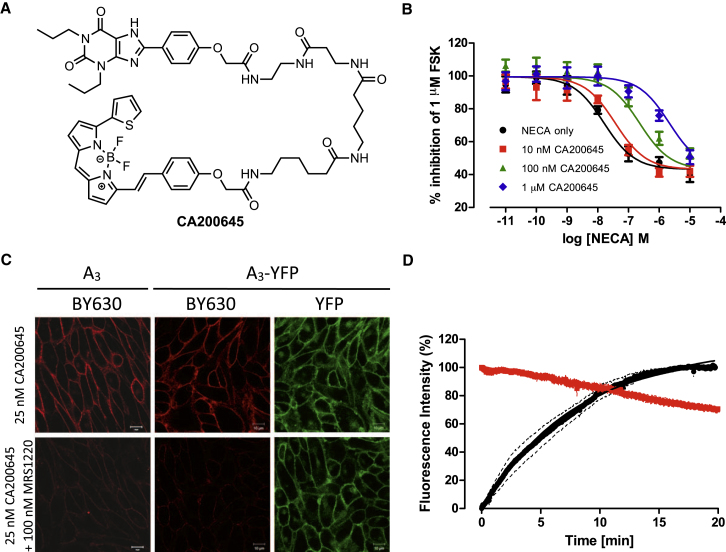
Pharmacologic Characterization of CA200645 (A) The fluorescent ligand CA200645. (B) Effect of increasing concentrations of the agonist, NECA, in the absence (•) and presence of 10 nM (▪), 100 nM (▲), and 1 μM (♦) CA200645 on the SPAP response to 1 μM FSK in cells expressing A_3_AR. Data are normalized to the response to 1 μM FSK and each data point represents mean ± SEM from three separate experiments performed in triplicate. (C) CHO cells expressing the A_3_AR or A_3_-YFP labeled with 25 nM CA200645, with and without preincubation with 100 nM MRS1220. Images shown are from a single experiment representative of three performed. (D) Association and dissocation kinetics of 25 nM CA200645. CHO A_3_AR cells were exposed to 25 nM CA200645 and confocal images were taken every 2 s for 20 min. Cells were washed with HBSS and images taken for a further 20 min. Data represents mean ± SEM from four separate experiments in which each replicate reflects the fluorescence intensity from the plasma membrane of ten cells. See also Figure S1.

CA200645 (CellAura Technologies Ltd) is a modification of an existing fluorescent xanthine amine congener (XAC) analog (Briddon et al., 2004) with a polyamide linker connected to the BY630 fluorophore (Figure 1A). To confirm that CA200645 retains antagonist properties at the A_3_AR, agonist concentration response curves were generated in the presence of CA200645 in Chinese hamster ovary (CHO) K1 cells stably expressing the human A_3_AR and a reporter gene consisting of a cAMP response element (CRE) promoter linked to a gene encoding secreted placental alkaline phosphatase (SPAP) (A_3_-CRE-SPAP cells). Measuring the levels of SPAP secreted from the cells gives an indirect measure of cAMP concentration within the cells and forskolin (FSK) is added to allow the A_3_AR mediated G_i/o_ response to be observed. Increasing concentrations of the agonist NECA resulted in a concentration-dependent reduction in SPAP production and incubation with increasing concentrations of CA200645 caused parallel rightward shifts in the NECA response curves (Figure 1B). Schild analysis revealed the logK_b_ of CA200645 at the A_3_AR to be −8.53 ± 0.10 (n = 3) with a Schild slope not significantly different from unity, indicating its action as a competitive antagonist.

Receptor-ligand interactions at the cell membrane were visualized using confocal microscopy. A_3_-CRE-SPAP cells were incubated with 25 nM CA200645 for 10 min at 37°C and single equatorial confocal images were obtained, which revealed clear CA200645 fluorescence at the cell membrane (Figure 1C, upper left panel). Specificity of the binding of CA200645 was established by pre-incubation of cells with the A_3_AR antagonist MRS1220 (100 nM, 30 min at 37°C), which resulted in a substantial reduction in the observed fluorescence (Figure 1C, lower left panel). Specificity of CA200645 binding was also confirmed using cells expressing the A_3_AR fused to yellow fluorescent protein (YFP; A_3_-YFP) (Figure 1C, middle panels) with high levels of colocalization seen between ligand and A_3_-YFP fluorescence. In both cases, very low levels of CA200645 fluorescence were seen in the cytoplasm, indicating that CA200645 mainly labels cell surface receptors and does not readily cross the cell membrane. To confirm this, A_3_-CRE-SPAP cells were incubated with 25 nM CA200645 for 2 hr at 37°C prior to imaging (Figure S1 available online), at which point clear membrane binding is still observed with little cytoplasmic fluorescence.

The kinetics of CA200645 binding to the A_3_AR were investigated using confocal microscopy performed at 37°C. CA200645 (25 nM) bound rapidly to A_3_-CRE-SPAP cell membranes, and was maximal by 15 min (Figure 1D). Cells were then washed twice with buffer and the dissociation of CA200645 monitored for a further 20 min at 37°C, after which ∼30% of ligand had dissociated from the cell membrane, indicating that CA200645 dissociates slowly from the A_3_AR (Figure 1D). The equilibrium dissociation constant of CA200645 calculated from this data (pK_D_ = 8.21 ± 0.12, n = 4) was similar to that obtained from functional assays.

### High-Content Screening Using a Confocal Imaging Plate Reader

**Figure 2:**
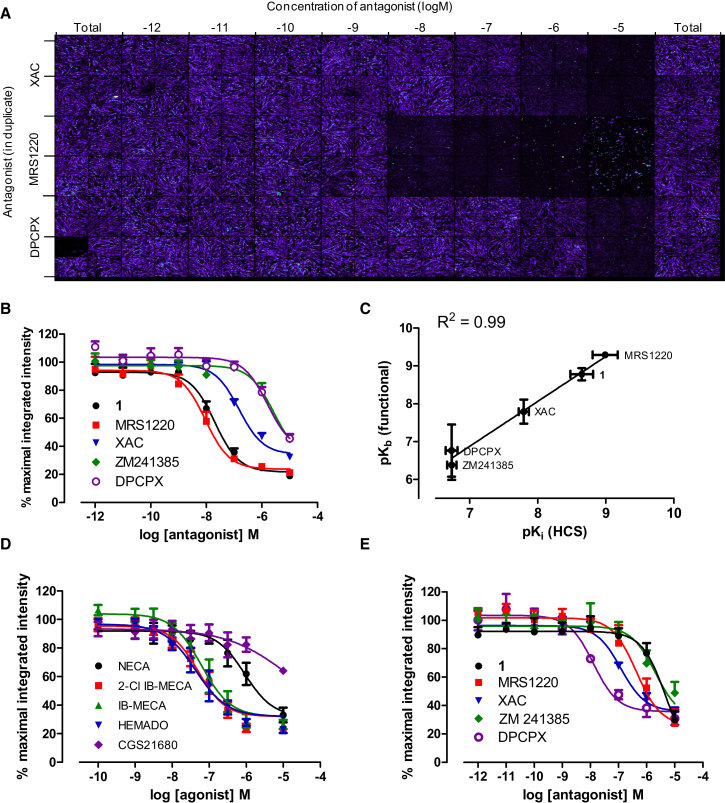
High-Content Screening Allows Competition Binding Curves to be Generated and Affinity Values Obtained at Both the A_3_AR and A_1_AR (A) Montage of one plate of images obtained using high-content screening in CHO CRE-SPAP A_3_AR cells. The two blank wells at the bottom of the first column are where the autofocusing of the plate was performed. (B) Competition curves at the A_3_AR generated from the total image intensity for five adenosine receptor antagonists. (C) Correlation between pK_i_ values obtained using HCS and pK_B_ values obtained in a functional SPAP assay. (D) Competition curves at the A_3_AR for five different adenosine receptor agonists. (E) Competition curves at the A_1_AR generated from the total image intensity in CHO-A_1_ cells for five adenosine receptor antagonists. Data are normalized to the maximal intensity observed per experiment and each data point represents the mean ± SEM from four (D and E) or five (B) experiments performed in duplicate. See also Figure S2.

The fluorescent A_3_AR antagonist CA200645 displayed many of the desired characteristics required for use in a high-content screening assay in live cells, including high affinity and a slow off-rate. A_3_-CRE-SPAP cells were grown in 96-well plates and incubated with increasing concentrations of three different antagonists with varying A_3_AR affinities, prior to addition of 25 nM CA200645. Subsequently, a confocal imaging plate reader (ImageXpress Ultra) was used for automated capture of four images per well. These images (Figure 2A) showed a clear decrease in membrane-bound CA200645 with increased competitor concentration. As expected, the high-affinity A_3_AR antagonist MRS1220 was able to displace CA200645 binding at lower concentrations than the moderate affinity antagonist XAC, while the A_1_AR specific antagonist DPCPX only displaced CA200645 binding at the highest concentration used (10 μM). Competition binding curves were generated using the mean of total image intensity from each well (Figure 2B), and pK_i_ values were calculated using the Cheng-Prusoff equation, using the K_b_ value for CA200645 (3.11 nM at A_3_AR) determined in the CRE-SPAP gene transcription assay (Table 1). The pK_i_ values obtained using the fluorescent ligand-based competition binding assay correlated well with the pK_b_ values obtained for the same compounds in the CRE-SPAP gene transcription assays including those with low affinity (R^2^ = 0.99; Figure 2C).

The assay was also able to quantify agonist displacement of CA200645 binding (Figure 2D), with three A_3_AR selective agonists, IB-MECA, 2-Cl IB-MECA, and HEMADO and the nonselective adenosine receptor agonist, NECA inhibiting CA200645 binding in a concentration-dependent manner (pIC_50_s = 7.13 ± 0.16, 7.13 ± 0.17, 7.23 ± 0.23, and 5.93 ± 0.15, respectively, n = 4) to levels similar to that observed in the presence of MRS1220. In contrast, only partial displacement of CA200645 binding was observed with the A_2A_AR selective agonist, CGS 21680.

CA200645 was also able to bind to the closely related human A_1_AR expressed in CHO cells (CHO-A_1_ cells; (Cordeaux et al., 2000) and antagonised calcium responses to NECA with a logK_b_ of −8.40 ± 0.15 (n = 4) (Figure S2). In an equivalent high-content, fluorescence-based binding assay for the A_1_AR, the affinities of competing ligands, calculated from the inhibition of CA200645 binding, were consistent with its known pharmacology (Figure 2E), with DPCPX having the highest affinity at the A_1_AR; and the A_3_AR selective antagonist triazolo-quinoxaline **1** (Lenzi et al., 2006), the lowest (Table 1).

Although data acquisition only took 20 min per plate, and the plate was read at room temperature using live cells, it was important to consider the potential for dissociation of CA200645 during this step. No significant dissociation of CA200645 was seen during image acquisition across the plate because the total membrane binding of CA200645 in the first column (Figure 2A, left side) was very similar to that in the last column (right side). Quantification of this binding over a number of experiments showed the total membrane intensity values for the last six wells read represented 96.7 ± 2.5% (n = 11) of the membrane intensities in the first six wells.

**Figure 3:**
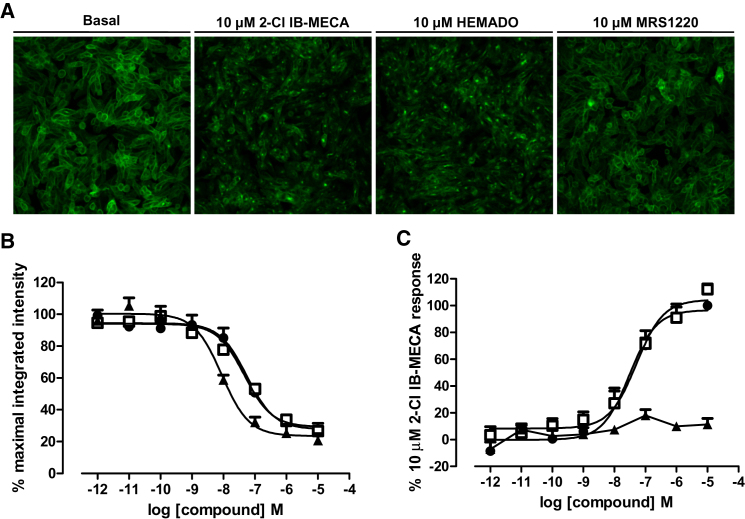
Simultaneous Measurement of Ligand Binding and Receptor Internalization Cells expressing A_3_-YFP were treated with increasing concentrations of the agonists HEMADO and 2-Cl IB-MECA and the antagonist MRS1220 for 30 min prior to the addition of 25 nM CA200645. CA200645 and YFP images were then obtained on the ImageXpress Ultra confocal plate reader after a further 30 min. Representative YFP images show that in the absence of ligand A_3_-YFP is clearly expressed at the cell surface and no change in localization is observed in the presence of MRS1220 (A). Clear redistribution of A_3_-YFP is observed in the presence of high concentrations of HEMADO or 2-Cl IB-MECA. Total image intensity for CA200645 images was obtained and granularity analysis was performed on YFP image. Competition curves were obtained with for MRS1220 (▪), HEMADO (□), and 2-Cl IB-MECA (•) (B) but only the agonists, HEMADO and 2-Cl IB-MECA, caused an increase in granule count (C). Data are normalized to the maximal intensity (B) observed or 10 μM 2-Cl IB-MECA response (C) per experiment and each data point represents the mean ± SEM from four experiments performed in duplicate.

Making use of data collection in two channels, it was also possible to use the high-content screening assay for simultaneous determination of ligand-binding and a functional response. Using CHO cells expressing the A_3_R tagged on its C terminus with YFP (CHO A_3_-YFP cells) simultaneous images of A_3_AR internalization and binding of CA200645 could be captured (Figure 3). Thus, two A_3_-selective agonists, 2-Cl IB-MECA and HEMADO, stimulated A_3_-YFP internalization, while the A_3_-selective antagonist MRS1220 did not (Figure 3A). Measurement of the binding of CA200645 30 min after addition of the above agents in the same wells confirmed that all three compounds inhibited CA200645 binding (Figure 3B). Quantification of A_3_AR internalization using granularity analysis (Kilpatrick et al., 2010) confirmed that only IB-MECA and HEMADO caused A_3_-YFP internalisztion (Figure 3C).

### Detection of Compounds Showing Low Binding Affinity

**Figure 4:**
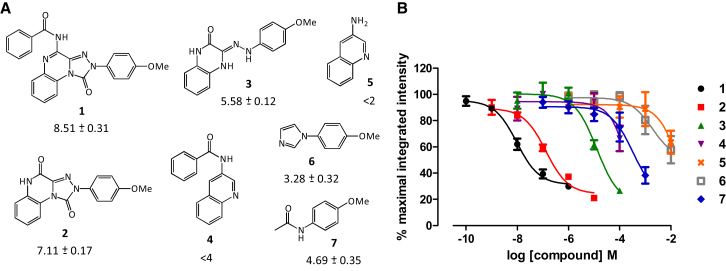
Displacement of CA200645 Binding by Fragments Demonstrates that a Wide Range of Affinities Can Be Determined at the A_3_AR (A) The high-affinity A_3_AR antagonist **1** was deconstructed into progressively lower molecular weight molecules **2–7**, and the pK_i_ values determined. (B) Competition curves at the A_3_AR generated from the total image intensity. Data are normalized to the maximal intensity observed per experiment and each data point represents mean ± SEM from at least three separate experiments performed in duplicate. See also Figure S3.

To determine if the high-content fluorescence-based competition binding assay could distinguish small molecules with low affinity at the A_3_AR, the high-affinity A_3_AR antagonist **1** was deconstructed into progressively smaller fragments **2–7** (Figure 4A). These compounds were tested for their ability to displace CA200645 binding at concentrations of up to 10 mM for the lowest molecular weight molecules (Figure 4). A wide range of affinities was measured for **2–7** (pK_i_ values from 7.11 to < 2), and the affinity of the fragments at the A_3_AR generally increased with molecular mass gain and increasing structural complexity. Small structural changes in some of the compounds resulted in relatively large shifts in potency. For example, there was a substantial increase in the binding affinity of quinoxaline-1,4-dione **2** (pK_i_ = 7.11 ± 0.17; n = 3) compared to the corresponding hydrazone **3** (pK_i_ = 5.58 ± 0.12, n = 3), presumably via a combination of conformational restriction and introduction of a new hydrogen bond acceptor.

### Screening of a Fragment Library

**Figure 5:**
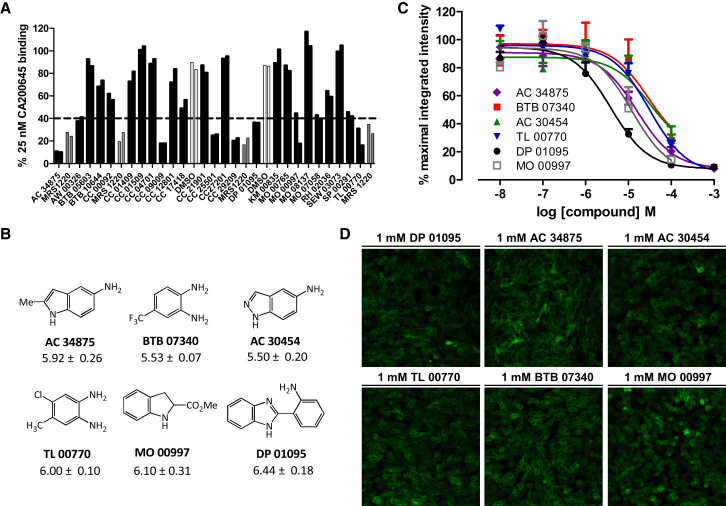
Screening a Commercial Fragment Library Identifies Hits with Affinities of Less Than 10 μM A total of 248 compounds from a Maybridge “Rule of Three” fragment library at a single concentration of 1 mM were tested for their ability to inhibit the binding of CA200645 at the A_3_AR. (A) A representative selection of the inhibition observed with 25 fragments and the controls, 1 μM MRS1220 (gray bars) and 1% DMSO (white bars). Any fragments that inhibited CA200645 binding to within 10% of that observed with MRS1220 were defined as hits and full concentration response curves defined as described in Figure 1. (B) Concentration response curves of the top six fragment hits at the A_3_AR. Each data point represents mean ± SEM from four separate experiments performed in duplicate. (C) The chemical structure of the top six fragment hits with mean ± SEM pK_i_ values shown below the structure. (D) Representative images from A_3_-YFP expressing cells treated with 1 mM for 1 hr at 37°C of the top six compounds. See also Tables S1 and S2.

To determine whether high-content imaging of living cells could be used to evaluate the binding of low-affinity compounds to the A_3_AR, a 248 compound subset from a Maybridge “Rule of Three” fragment library were tested for their ability to inhibit the binding of CA200645 to the A_3_AR at a single concentration of 1 mM (Figure 5). Figure 5A shows a representative selection of 25 fragments with differing affinities for the receptor. Any compound that inhibited CA200645 binding to within 10% of that observed with 1 μM MRS1220 was taken forward for full concentration analysis (Table S1). The pK_i_ values for all 38 of these compounds are given in Table S2 and the data and structures for the six most potent fragments are shown in Figures 5B and 5C. DP 01095 was the most potent fragment from this screen with a pK_i_ value of 6.44 ± 0.18. Interestingly, pK_i_ values as low as 3.97 could be measured for these fragments (Table S2). Using CHO A3-YFP cells as described previously, it was confirmed that none of the fragments that underwent full concentration analysis acted as agonists at the A_3_AR. Respresentative images from cells treated with the six most potent compounds are shown in Figure 5D.

### Chemical Modification of a Lead Fragment to Provide A_3_-Receptor Selectivity

**Figure 6:**
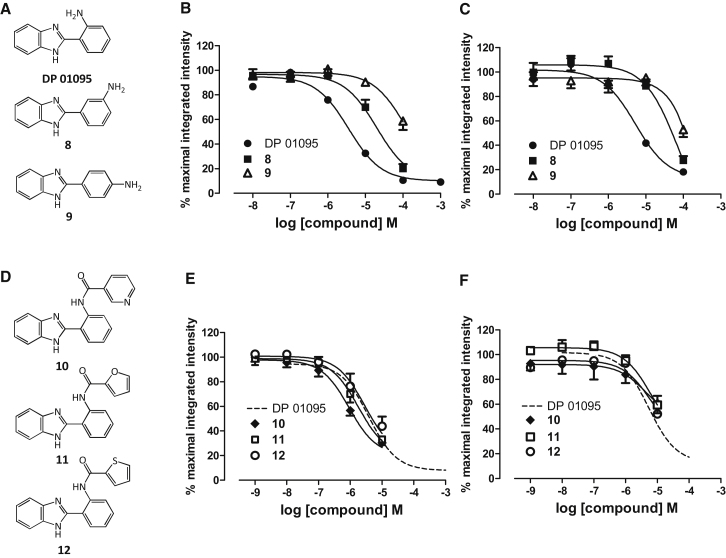
Elaborated Derivates of DP 01095 Show Higher Affinity at the A_3_AR but Not at the A_1_AR (A) Structure of the top fragment hit (DP 01095) and two derivatives, **8** and **9**. (B and C) These fragments were tested for their ability to inhibit the binding of CA200645 in cells expressing the A_3_AR (B) or A_1_AR (C). Each data point represents mean ± SEM from six (B) and four (C) experiments performed in duplicate. (D) Structure of three synthesized DP 01095 derivatives (**10–12**). (E and F) The ability of these three compounds to inhibit the binding of 25 nM CA200645 in cells expressing the A_3_AR (E) or A_1_AR (F) receptor is shown. The dashed lines show the curve obtained with DP 01095. Each data point represents mean ± SEM from four experiments performed in duplicate. See also Figure S4 and Table S3.

Having identified DP 01095 as the most potent fragment, its structure lent itself to address whether our assay was sensitive enough to differentiate any early structure activity relationship (SAR) profiles and/or receptor subtype selectivity through further expeditious chemical elaboration. We therefore initially synthesized two alternative structural isomers based on the position of the anilino amine (Figure 6A) and assayed these and the parent compound at both the A_1_AR and A_3_AR. For both receptor subtypes the three isomers demonstrated a similar rank order of affinity (Figures 6B and 6C); DP 01095 remained the highest affinity A_3_AR compound (pK_i_ = 6.48 ± 0.14) followed by the 3-aminophenyl **8** (pK_i_ = 5.49 ± 0.11), with the *para*-derivative **9** (41.2 ± 7.3% inhibition at 100 μM) displaying the weakest affinity. Because DP 01095 remained the highest affinity lead fragment for the A_3_AR, we elaborated this molecule via acylation or alkylation of the anilino nitrogen to afford a focused 14-member mini-library (compounds **10–23**, Table S3; for synthetic procedures, see Supplemental Experimental Procedures). Initial screening against the A_3_AR resulted in an identifiable SAR with the acylated anilines (**10–16**) displaying the largest inhibition of fluorescent ligand binding when compared to the alkylated derivatives **17–23** (Table S3). Only five compounds were capable of inhibiting binding by >50% and a pK_i_ was derived for these compounds (Table S3). The 3-nicotinoyl amide derivative **10**, displayed a 3-fold increase in affinity when compared to DP01095 (Figure 6E), while only the furan 2-carboxamido (**11**) and thiophene 2-carboxamido (**12**) derivatives showed enhanced A_3_AR affinity compared to DP 01905. Rewardingly, the enhancement of binding observed for **10**, **11**, and **12** was not mirrored at the A_1_AR (Figure 6F), where all three ligands inhibited binding by less than 50% at 10 μM. This confirmed that acylated heteroaryl derivatives **10–12**, unlike the DP 01095 precursor, displayed measurable A_1_AR/A_3_AR subtype selectivity.

## Discussion

It is now well established that GPCRs can adopt multiple conformations to interact with different signaling pathways and this can lead to the phenomenon of ligand bias (Kenakin and Miller, 2010; Kenakin, 2012; Williams and Hill, 2009). It is thus a major challenge to develop multiwell plate assays for the measurement of ligand affinity in living cells where the receptor is in the correct cellular context, genetically identical to the native human receptor and where the integrity of its local membrane environment is maintained. Here, we have succeeded in demonstrating that a fluorescent XAC derivative, CA200645, can be used in conjunction with a confocal imaging plate reader to provide an appropriate assay that allows receptor characterization, fragment screening and the evaluation of low-affinity compounds in living cells expressing human A_3_ and A_1_ receptors.

The A_3_AR has been implicated as a drug target in a number of pathophysiologic conditions including cancer, ischemia, cardiovascular disease, and inflammation. Historically, the affinity of a ligand for a target receptor is most commonly determined by measuring the ability of an unlabelled ligand to compete with a single concentration of radiolabelled ligand at equilibrium in isolated membrane preparations (Hulme and Trevethick, 2010). An antagonist radioligand is the preferred probe of choice because it avoids the complication of differential labeling of active (R^∗^) and inactive (R) receptor conformations by radiolabelled agonists (Clark and Hill, 1995; Cordeaux et al., 2008). However, radiolabelled antagonists for the A_3_AR have been reported but are not available commercially (Varani et al., 2000). Radiolabelled ligands have the advantage that the incorporation of a radioisotope into a ligand does not affect its chemical structure, therefore assays to measure their binding are relatively easy to set up and optimize. However, the final assay read-out is not in real time and the assays are often conducted in disrupted membrane preparations where cellular integrity has been lost. This can have a major impact on binding characteristics as a consequence of buffer composition, loss of membrane potential, and the presentation of buffer components and drugs to both sides of the cell membrane. In addition, the safety risks associated with handling radioligands in large screening campaigns (particularly those based on ^125^I-labeled ligands) and the associated legal and disposal requirements make alternatives highly preferred.

Here, we show that the fluorescent XAC derivative, CA200645, is a high-affinity adenosine receptor antagonist that binds specifically to cell-surface expressed A_1_AR and A_3_ARs. Its slow off-rate from the receptors allowed imaging (at room temperature) to take place following washout of the fluorescent probe. These properties allowed a competition binding assay to be developed in living cells based on measuring total image intensity from confocal images captured and analyzed automatically, which gave accurate and precise affinity values for known ligands at the A_3_AR. We have shown that this method can resolve a wide range of affinity values and identify subtle differences in antagonist affinities. CA200645 also had a high affinity for the A_1_AR expressed in CHO cells, allowing the same assay format to be used to evaluate the selectivity of competing ligands for A_1_AR and A_3_AR. It was notable that the selective A_3_AR and A_1_AR antagonists, MRS 1220 and DPCPX, respectively, showed the appropriate affinity profiles in these two receptor systems. By using a high-affinity fluorescent antagonist such as CA200645, we show that a fluoresecent competition binding assay can be developed that directly detects the ligand. This removes the need for genetic modification of the receptor, which is a requirement of other GPCR ligand-binding assays that use fluorescent ligands (Zwier et al., 2010), and would potentially allow the assay to be used directly on cells and tissues that endogenously express the A_1_AR and A_3_AR.

As mentioned, fragment-based assays are powerful tools in drug discovery to explore chemical space more effectively. However, the majority of previous work on GPCRs has involved highly purified detergent solubilized receptors where removal of the protein from its native cellular context may isolate it from the normal allosteric influences of intracellular signaling and other associated membrane proteins it experiences in living cells. There are now a significant number of privileged molecular scaffolds that have been reported as possessing significant A_3_AR subtype selectivity (Figure S3). The development of a live cell ligand-binding assay for the A_3_AR allowed us to evaluate whether fragment screening could be undertaken in an intact cellular environment. From a library of 248 molecules (Maybridge “Rule of Three”), we were indeed able to assay and identify a range of fragments that displayed binding affinities in the micromolar range.

Analysis of the hits from our screen allowed us to ascertain if we had successfully identified compounds containing structural motifs present within established A_3_-selective ligands (Figure S3). For example, the 1,3-thiazole nucleus has been recently confirmed as a key component in many adenosine receptor antagonists (Figure S4A and associated references). Of the 248 compounds assayed, there were 20 examples of this substructure within the library (two of which were embedded in a benthiazole fragment). Of the 18 non-ring fused thiazole variants, our assay identified five as hits (Figure S4A). However, modeling studies performed to-date suggest that the key receptor-ligand interactions are achieved through the substituents present on the heterocyclic ring rather the thiadiazole itself (Miwatashi et al., 2008). One could therefore envisage that with such simple fragments, binding via alternative geometries is entirely probable and it was encouraging that the more highly decorated heteroaryl-aryl linked hit fragments did indeed possess the superior binding affinities; possible by virtue of additional points of contact with the receptor binding site. More rewarding, however, was the observation that our assay identified the only 1,2,4-thiadiazole present within the library and this overlaid well with recently established literature compounds (Jung et al., 2004; van Muijlwijk-Koezen et al., 2001) (Figure S4B). Indeed, simple acylation of the fragment's aromatic amino group would naturally arrive at the nanomolar affinity compounds illustrated in the literature. Our assay also identified the sole quinazoline fragment within the library as a hit. In addition, we were also able to identify three of the four pyrimidines present. Crucially this included the only example of a 2-phenylpyrimidine; a scaffold recently identified as providing the basis of potent A_3_AR antagonists (Figure S4C) (Yaziji et al., 2011). Taken together, these and other fragments identified from our screen serve as excellent exemplars that this fluorescence-based screening approach could indeed generate important lead compounds in a drug discovery program.

Ultimately however, from our library of 248 molecules we identified our favored lead fragment DP 01095 with high affinity (pK_i_ = 6.44), which provided a chemical template for the design of A_3_AR antagonists. 1*H*-Benzimidazol-2-yl systems have been reported as antagonists of the A_1_AR, however with quinoline and isoquinoline substitutents at the 2-position (Cosimelli et al., 2011). The structure of DP 01095 was ideal to assess whether our assay was sensitive enough to allow identification of early SAR profiles and/or receptor subtype selectivity through further chemical modification. A decrease in affinity was observed as the anilino amine was relocated onto the *meta-* (**8**) and *para*- (**9**) positions. The observed rank order of affinity at the A_3_AR was *ortho > meta > para* and this SAR transposed onto the A_1_AR, albeit with a minor yet concomitant reduction in ligand affinities. Encouraged by these results, we undertook further structural refinements through either acylation (**10–16**) or alkylation (**17–23**) of the anilino amine of DP 01095 to afford a focused 14-member mini-library of alkyl, aryl, and heteroaryl functionalised benzimidazoles. The three heteroaryl carboxamides (**10–12**) that displayed increased binding affinities for the A_3_-receptor compared to DP 01095 were all demonstrated to exhibit a much lower affinity for the A_1_-receptor. These data suggest that confocal imaging based assays of fluorescent ligand binding to transmembrane receptors expressed on whole living cells, in combination with the data becoming available on the crystal structure of a wide range of GPCRs, has the potential to revolutionise the drug discovery process involving this receptor family.

## Significance

**Development of new approaches that allow the screening of both drug and fragment-like molecules at GPCRs in a live cell format would be a significant addition to the drug discovery process. We describe the development of a robust competition binding assay for two such GPCRs, the adenosine-A_1_ and -A_3_ receptors, which use a fluorescent antagonist ligand. This assay uses automated capture and analysis of confocal images in a multiwell format to obtain quantitative information on ligand-binding parameters in living cells, allowing accurate determination of affinity values for literature antagonists. This assay has advantages over currently described methodology because it is does not require modification of the receptor of interest and is ammeanable to high throughput screening. We demonstrated that fragment screening can be carried out on living cells and that a viable chemical lead could be indentified. The highest affinity fragment had a dissociation constant of less than 1 μM and, to our knowledge, possessed a novel structure for an AR antagonist. A small library of compounds based on this lead fragment was synthesized and a higher affinity A_3_AR antagonist was found which had little affinity at the A_1_AR. High-throughout fragment screening using living cells could address one of the major issues in the GPCR drug discovery process because there is no need for receptor purification and biophysical approaches.**

## Experimental Procedures

### Chemicals and Synthesis

All transfection reagents and G418 were obtained from Invitrogen. Fetal calf serum was obtained from PAA Laboratories and L-glutamine from Lonza. MRS1220, DPCPX, ZM241385, IB-MECA and HEMADO were purchased from Tocris Bioscience. All other biological reagents were obtained from Sigma-Aldrich. CA200645 was obtained from CellAura Technologies. General chemistry methods and the synthesis of **1** to **23** are described in the Supplemental Information.

### Cell Culture and Generation of Stable Cell Lines

CHO-K1 cells stably expressing a cAMP response element-secreted placental alkaline phophatase (CRE-SPAP) reporter gene (Baker et al., 2002) and CHO-K1 cells stably expressing the human A_1_AR were maintained in DMEM/F12 medium containing 10% fetal calf serum and 2 mM L-glutamine at 37°C in a humidified atmosphere of air/CO_2_ (19:1). To generate stable cell lines, CHO CRE-SPAP cells were transfected with the pcDNA3.1 plasmid containing cDNA encoding the full length human A_3_AR or the human A_3_AR that was fused in-frame with YFP (A_3_-YFP) using Lipofectamine according to manufacturer's instructions. Transfected cells were subjected to selective pressure for 2 to 3 weeks through the addition of 1 mg/ml G418 to the normal growth medium. Cells were dilution-cloned to obtain near-clonal cell lines. Cell lines expressing A_3_-YFP were first screened for YFP fluorescence. Cell lines (A_3_AR and A_3_-YFP) were then screened for a response in the CRE-SPAP gene transcription assay.

### CRE-SPAP Gene Transcription Assay

CHO CRE-SPAP cells stably expressing the A_3_AR were grown to confluence in 96-well plates and on day prior to analysis, normal growth medium was removed from the cells and replaced with serum free medium (DMEM/F12 containing 2 mM L-glutamine). On the day of analysis, the medium was removed and replaced with fresh serum free medium containing the required concentration of CA200645 and cells incubated for 1 hr at 37°C/5%CO_2_. After 30 min, the required concentration of the agonist NECA was added and cells incubated for further 30 min at which time 1 μM forskolin was added. Cells were incubated for 5 hr at 37°C/5% CO_2_. Following the 5 hr incubation, SPAP quantification was performed as described by Baker et al. (Baker et al., 2002).

### Confocal Microscopy

For single images, CHO CRE-SPAP cells stably expressing the A_3_AR or A_3_-YFP were grown in Nunc Labtek 8-well plates, and images obtained using a Zeiss LSM5 Exciter confocal microscope fitted with a 63x plan-Apochromat 1.4NA oil-immersion DIC objective at 37°C. A 488 nm argon laser was used to excite YFP and emission detected using a BP505-530 filter. BY630 was excited using a 633 nm HeNe laser and emission detected using a LP650 filter. Prior to imaging, normal growth medium was removed and cells were washed twice in HEPES-buffered saline solution (HBSS; Briddon et al., 2004) and fresh HBSS added for analysis. Cells were incubated with 25 nM CA200645 for 10 min at 37°C before imaging. The specificity of CA200645 binding was determined by pre-incubating cells with 100 nM MRS1220 for 30 min at 37°C prior to the addition of CA200645.

To determine the kinetics of CA200645 binding, cells were grown on 22 mm circular coverslips and imaged using a Zeiss LSM 510 confocal microscope using a 40× 1.3 NA oil-immersion objective (May et al., 2010). Coverslips were mounted in an imaging chamber in HBSS, 25 nM CA200645 added and fluorescence and phase images collected every 2 s for 20 min. BY630 was excited using a 633 nm HeNe laser and emission detected using a LP650 filter. After 20 min, cells were washed three times with HBSS, then fresh HBSS added to the cells and images collected for a further 20 min. The change in fluorescence intensity values were obtained on a single cell basis by drawing a region of interest around the plasma membrane of ten cells per field using the Zeiss AIM 4.2 software.

### High-Content Competition Binding Assay

CHO CRE-SPAP cells stably expressing the A_3_AR or A_3_-YFP or CHO A1 cells were seeded into the central 60 wells of a 96-well clear-bottomed, black-walled plate and grown to confluency. On the day of analysis, normal growth medium was removed and cells washed twice with HBSS pre-warmed to 37°C. Fresh HBSS was added to each well and the required concentration of unlabelled compound added. The cells were incubated at 37°C for 30 min, then 25 nM CA200645 was added to each well and the cells incubated for a further 30 min at 37°C/5% CO_2_. Buffer was then removed from each well, cells washed once in HBSS and fresh HBSS added at room temperature. Plates were immediately imaged using an ImageXpress Ultra confocal plate reader which captured four central images per well using a Plan Fluor 40x NA0.6 extra-long working distance objective. CA200645 was excited at 635 nm and emission collected through a 640-685 nm band pass filter while YFP was excitated at 488 nm with emission collected through a 525-550 nm band pass filter. Total image intensity was obtained using a multi-wavelength cell scoring algorithm within MetaXpress software (MetaXpress 2.0, Molecular Devices). Granularity analysis was performed on YFP images using a granularity algorithm also within MetaXpress software and granules were defined as having a diameter of 7–15 μm as previously described (Kilpatrick et al., 2010).

### Commercial Fragment Library Screen

From the “Rule of Three” Fragment Library (Maybridge, UK), 248 compounds were chosen by refining for a molecular weight of 100–350 Da, hydrogen bond donors 0–3, hydrogen bond acceptors 1–3, TPSA between 10 and 60 Å^2^ and rotatable bonds 0–4. DMSO-*d*_*6*_ as was added to the compound (5 mg) to achieve a concentration of 100 mM. Compounds were diluted to 10 mM in HBSS and any compound that formed an obvious precipitate was not screened. Compounds were screened at an initial concentration of 1 mM and it was found that the high-content competition binding assay was tolerant of concentrations of DMSO up to 1%. After initial screening, the purities of the top 38 compounds were determined. This quality control analysis was performed using a Shimadzu 2020 LCMS system, a Waters Sunfire C18 column (3.5 μm, 2.1 mm × 30 mm), and Shimadzu Lab solutions software. Compounds were analyzed by integration of the chromatogram peak at 254 nm and 220 nm that corresponded to the designated compound mass, and were shown to be ≥ 95% pure at each wavelength.

### Data Analysis

All data were fitted using Prism 5 (GraphPad Software). Data analysis for estimating pK_B_ values from the CRE-SPAP gene transcription, calcium mobilization assay and the kinetic analysis of CA200645 binding can be found in Supplemental Experimental Procedures. All competition binding curves with the fluorescently labeled antagonist were fitted to the following equation to calculate the binding affinity (K_i_) of the ligand to the receptor:

$$K_{i}=\frac{IC_{50}}{1+\frac{\left[L\right]}{K_{D}}},$$

where [L] is the concentration of CA200645 in nM, K_D_ is its K_D_ in nM calculated from functional assays and the IC_50_ is calculated from the following equation:

$$\%\;\text{inhibition}\;\text{of}\;\text{specific}\;\text{binding}=\frac{100\times \left[A\right]}{\left[A\right]+IC_{50}},$$

where [A] is the concentration of competing drug and the IC_50_ is the molar concentration of the ligand required to generate 50% inhibition of specific binding.

## Figures and Tables

**Table 1:** Binding Affinities of Literature Antagonists against the Adenosine A_3_AR and A_1_AR

	A_3_AR				A_1_AR	
	Fluorescent Ligand-Binding Assay^a^		Functional SPAP Assay^b^		Fluorescent Ligand-Binding Assay^a^	
	pK_i_	n	pK_B_	n	pK_i_	n
**1**	8.51 ± 0.31	6	8.78 ± 0.16	3	5.93 ± 0.12	3
MRS1220	9.02 ± 0.22	6	9.29 ± 0.08	6	7.14 ± 0.38	3
XAC	7.85 ± 0.14	6	7.79 ± 0.32	6	7.54 ± 0.10	4
DPCPX	6.96 ± 0.02	6	6.76 ± 0.69	3	8.54 ± 0.17	4
ZM 241385	6.74 ± 0.07	5	6.39 ± 0.65	3	6.68 ± 0.11	3
PSB 603	7.25 ± 0.11	4	ND		7.38 ± 0.02	4
CGS 15943	8.18 ± 0.10	4	ND		8.95 ± 0.08	4
SCH 58261	6.73 ± 0.26	4	ND		7.29 ± 0.08	4

a Values obtained in a fluorescent adenosine receptor antagonist binding assay using whole, live cells expressing the A_1_AR or A_3_AR.

b Measured by the shift of NECA-mediated inhibition of forskolin stimulated CRE-SPAP response in CHO CRE-SPAP cells expressing the A_3_AR. Values represent mean ± SEM from n separate experiments and ND = not determined.

## Supplemental Information

Document S1. Supplemental Experimental Procedures, Figures S1–S4, and Tables S1–S3
