# Supplementary material for: Fragment Screening at Adenosine-A3 Receptors in Living Cells Using a Fluorescence-Based Binding Assay
Source: Chem Biol. 2012 Sep 21;19(9):1105–15. doi: 10.1016/j.chembiol.2012.07.014 (PMC3456874; doi:10.1016/j.chembiol.2012.07.014)
Supplement: Document S1. Supplemental Experimental Procedures, Figures S1–S4, and Tables S1–S3 [file mmc1.pdf]

Chemistry & Biology, Volume *19*

Supplemental Information

Fragment Screening
at Adenosine-A_3 Receptors in Living Cells Using
a Fluorescence-Based Binding Assay

Leigh A. Stoddart, Andrea J. Vernall, Jessica L. Denman, Stephen J. Briddon, Barrie Kellam, and Stephen J. Hill

Inventory of Supplemental Information

Supplemental Experimental Procedures and Data Analysis S3

Figure S1: Membrane binding of CA200645 can still be observed after 2h incubation

- **related to Figure 1 S6**

Figure S2: CA200645 as a high affinity antagonist at the adenosine A_1 receptor

- **related to Figure 2 S7**

Figure S3: A selection of reported adenosine A_3-receptor selective antagonists

- **related to Figure 4 S8**

Figure S4. Analysis of fragment screen hits in relation to established adenosine receptor ligands

- **related to Figure 6 S9**

Table S1: Inhibition of CA200645 binding by Maybridge "Rule of Three" library

- **related to Figure 5 S11**

Table S2: Binding affinities of 38 Maybridge "Rule of Three" fragments

- **related to Figure 5 S13**

Table S3: Binding affinities of DP01095 derivates at the adenosine A_3 and A_1 receptors

- **related to Figure 6 S14**

General chemistry methods S16

Synthesis and characterization of compounds 1 – 7 S17

Synthesis and characterization of compounds DP 01095, 8, and 9 S20

Synthesis and characterization of compounds 10 - 16 S22

Synthesis and characterization of compounds 17 - 23 S26

Supplemental references S30

Supplemental Experimental Procedures

Calcium mobilization assay.

CHO cells expressing the adenosine A_1 receptor were grown to confluence in black-walled, clear-bottom 96-well plates. On the day of the experiment, medium was removed from each well and replaced with 100 µL HEPES-buffered saline solution (HBSS; 25 mM HEPES, 10 mM glucose, 146 mM NaCl, 5 mM KCl, 1 mM $MgSO_4$, 2 mM sodium pyruvate, 1.3 mM $CaCl_2$, 1.5 mM $NaHCO_3$) supplemented with 2.5 mM probenecid, 2.3 µM Fluo-4AM and 0.023% Pluronic F-127. Cells were incubated with Fluo-4 containing buffer for 45 min at 37°C in the dark. Cells were then washed twice in HBSS and fresh HBSS with 2.5 mM probenecid with or without CA200645 added to each well and cells incubated for a further 30 min at 37°C in the dark. Plates were then loaded onto a multi-well fluorimetric imaging plate reader (FlexStation; Molecular Devices, Sunnyvale, CA) and fluorescence was measured (excitation, 485 nm; emission, 520 nm) every 1.52 s for 200 s. Either HBSS or HBSS with the required concentration of NECA was added at 15 s).

Data Analysis. For the CRE-SPAP gene transcription assay, NECA concentration-response curves in the absence and presence of increasing concentrations of test compounds were globally fit to the following interaction model in Prism5.

$$\mathrm{Response} = \frac{\mathrm{E_{max}} \times [\mathrm{A}]}{([\mathrm{A}] + \mathrm{EC_{50}} \times \left(1 + \frac{[\mathrm{B}]}{\mathrm{K_B}}\right)^{S})}$$

Where E_{max} is the maximal response, EC_{50} is the molar concentration of NECA [A] in the absence of test compound required to generate a response that is 50% of E_{max}, [B] is the concentration of test compound, K_D is the antagonist equilibrium dissociation constant and S is the Schild slope. For the

calcium mobilization experiments, estimated affinity values (pK_B) were calculated from the shift of the agonist concentration response curves elicited in the presence of the CA200645 using the following equation:

$$DR = 1 + \frac{[B]}{K_B}$$

Where DR (dose ratio) is the ratio of the agonist concentration required to stimulate an identical response in the presence and absence of antagonist, [B]. As there was a decrease in observed maximal efficacy of NECA in the presence of CA200645, the EC_{25} value was used to determine the DR.

The association and dissociation kinetic analysis of CA200645 binding to A_3AR was simultaeneously to the following equations:

$$Y = Y_o + (Plateau - Y_o)(1 - e^{-k_{onobs} .t})$$

and

$$Y = (Y_o - Plateau).e^{-k_{off} .t} + \text{Plateau}$$

where Y_o is the level of CA200645 binding at time (t) zero, Plateau is the level of CA200645 binding at infinite time, k_{onobs} is the observed rate of association and k_{off} represents the rate of CA200645 dissocaition. To calculate the association rate (k_{on}) and the negative logarithm of the equilibrium dissociation constant (pK_D) from k_{on} and k_{off} the following equations were used:

$$k_{on} = \frac{k_{onobs} - k_{off}}{[CA200645]}$$

and

$$pK_D = -\log\left(\frac{k_{off}}{k_{on}}\right)$$

where k_{onobs}, k_{on} and k_{off} are as described above.

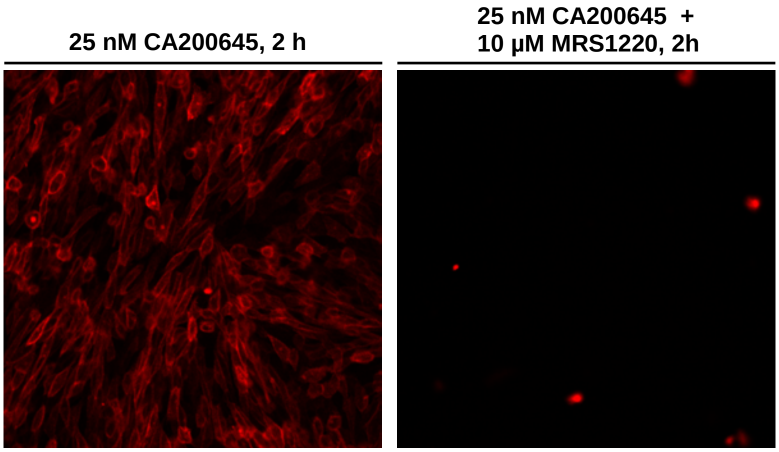

Figure S1. Membrane binding of CA200645 can still be observed after 2h incubation

CHO CRE-SPAP cells expressing the A_3AR receptor were incubated with 25 nM CA200645 in HBSS (left hand panel) or 25 nM CA200645 and 10 µM MRS1220 (right hand panel) for 2 h at 37°C. Cells were washed twice in fresh HBSS and images captured automatically using the Ultra confocal plate reader. After 2h, clear membrane binding of CA200645 is still idenfiable, with little fluorescent observed within the cells and this is completely blocked in the presence of MRS1220. Images are respresentative of four experiments performed in duplicate.

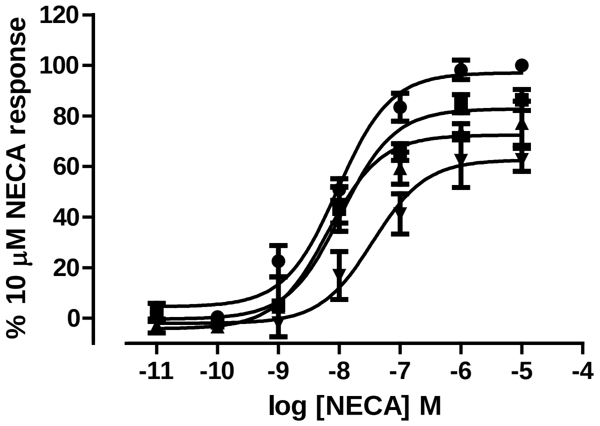

Figure S2. CA200645 as a high affinity antagonist at the adenosine A_1 receptor. CHO cells expressing the adenosine A_1 receptor were stimulated with increasing concentrations of NECA (●) and the resulting increase in intraceullar calcium measured via Fluo4. The presence of 10 nM (■), 30 nM (▲) and 100 nM (▼) CA200645 caused a shift in the agonist concentration response curve showing that CA200645 but also a depression of the maximum response therefore the method described by Christopoulos et al. (1999) was used to calculate the apparent pK_b of the antagonist which is 8.29 ± 0.16. Each data point represents mean ± s.e.m. of four experiments performed in triplicate.

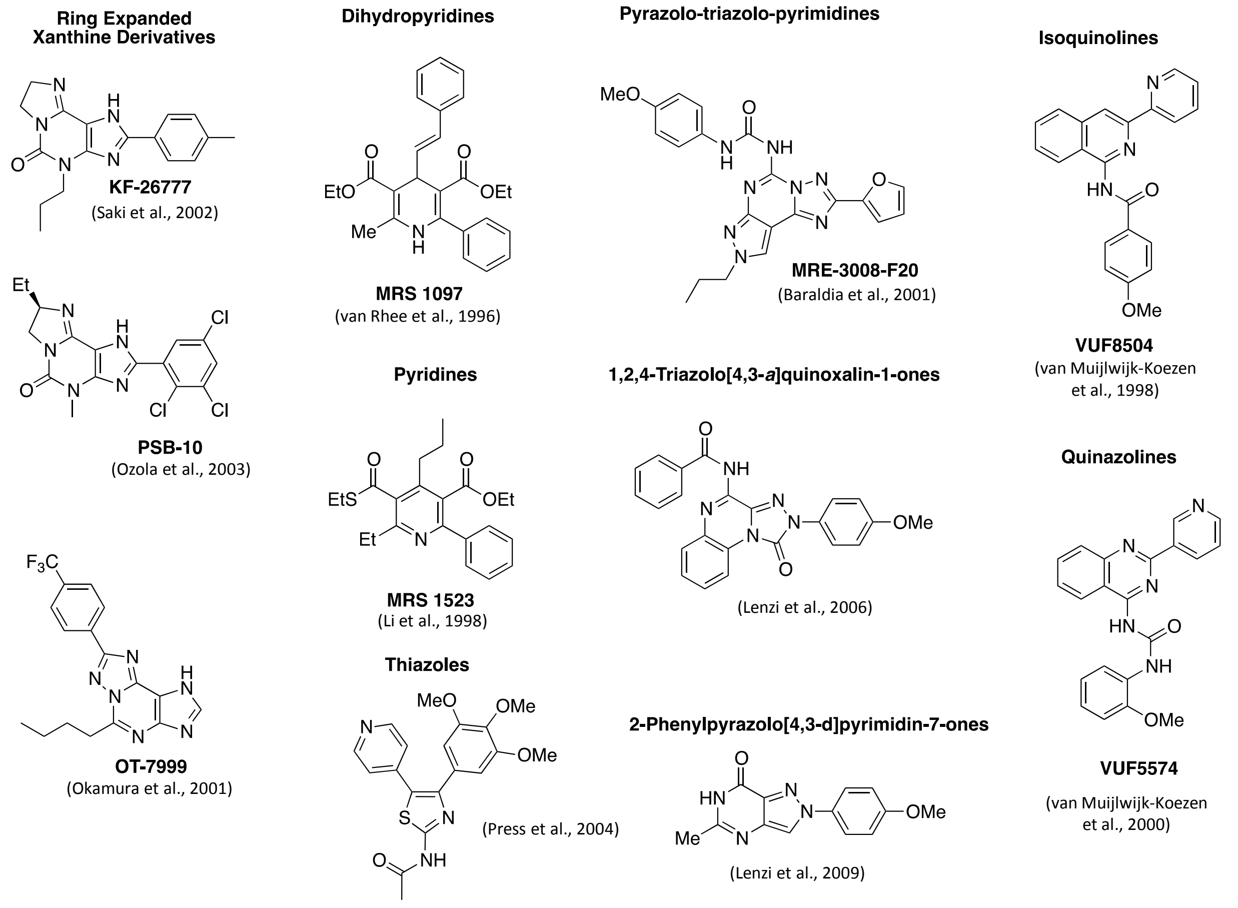

Figure S3. A selection of reported adenosine A_3-receptor selective antagonists..

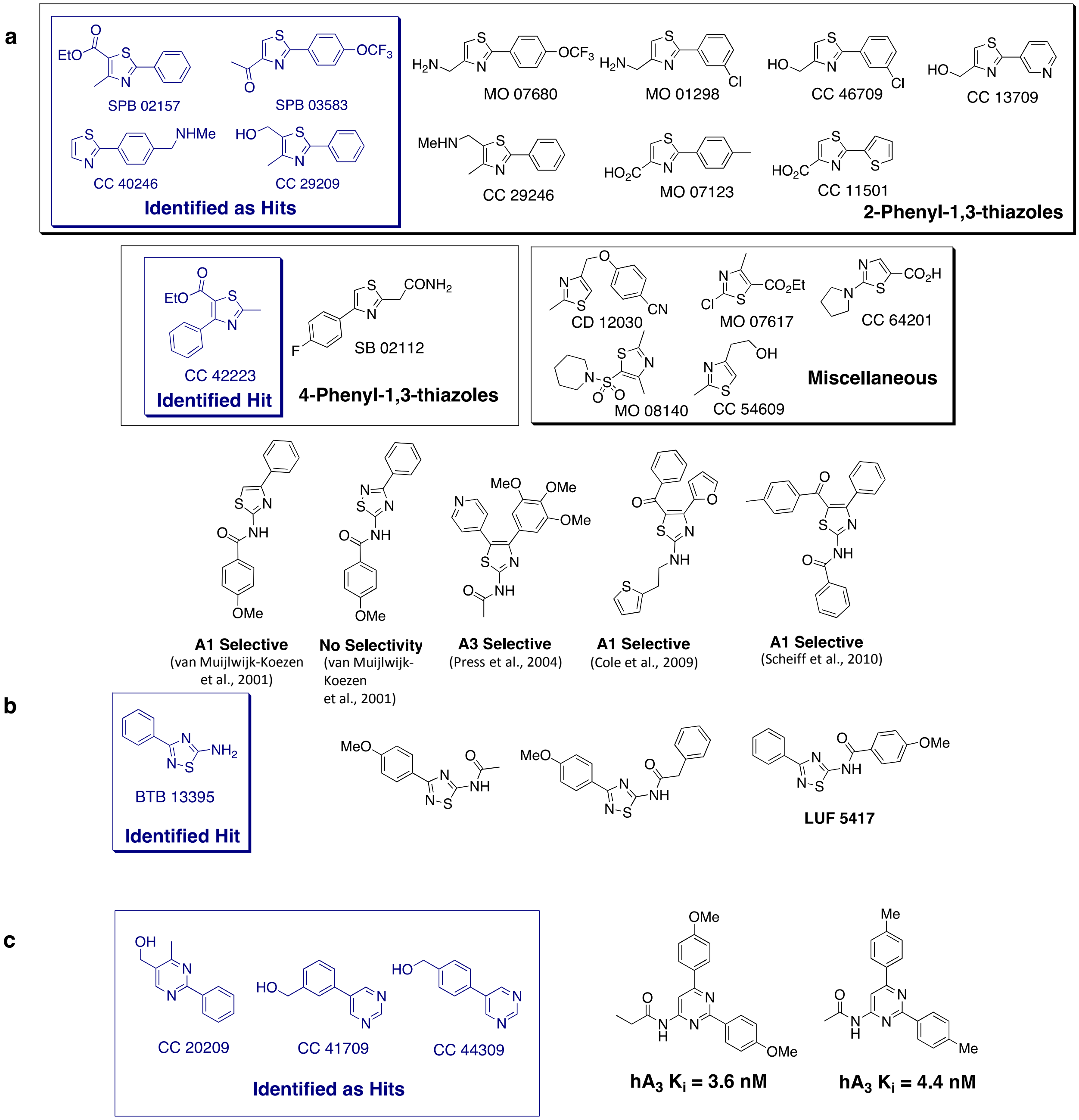

Figure S4. Analysis of fragment screen hits in relation to established adenosine receptor ligands.

(a) 18 Thiazole-containing fragments were contained within the 248-member library screened using our live-cell assay. Four out of eleven 2-phenyl-1,3-thiazoles were identified as hits, alongside one of the two 4-phenyl-1,3-thiazoles. The thiazole and thiadiazole ring systems have been recently identified in numerous adenosine receptor antagonists, although there is no direct fragment correlation with any of

the Maybridge Library members we assayed. (b) There was one 1,2,4-thiadiazole member of the 248-member library and this compound, BTB 13395, was identified as a hit. The fragment maps well onto a range of established adenosine A_3-receptor antagonists recently described in the literature (Jung et al., 2004; van Muijlwijk-Koezen et al., 2001). (c) Fragment CC 20209, the only 2-phenylpyrimidine member of the assayed 248-member library, was successfully identified as a hit. The fragment maps well onto a range of established adenosine A_3-receptor antagonists recently described in the literature (Yaziji et al., 2011).

Table S1. Inhibition of CA200645 binding by a library of fragments from a Maybridge "Rule of Three" library. Values obtained in a fluorescent adenosine receptor antagonist binding assay using whole, live cells expressing the adenosine A_3 receptor. Values quoted are % of control wells (wells containing 1% DMSO and 25 nM CA200645). All compounds were tested at 1 mM. Compounds selected for further screening are indicated by an asterisk (*) and when CA200645 binding was within 10% of the inhibition observed with the control compound (1 µM MRS1220) included in each plate.

Maybridge compound code	% of CA200645 binding (% DMSO control)		Maybridge compound code	% of CA200645 binding (% DMSO control)		Maybridge compound code	% of CA200645 binding (% DMSO control)		Maybridge compound code	% of CA200645 binding (% DMSO control)
AC 1258	95.05		CC 12701	58.41		CC 49909	61.21		MO 08137	110.72
AC 30454	13.01*		CC 12801	77.99		CC 50209	57.90		MO 08148	108.00
AC 34875	10.43*		CC 13209	66.20		CC 50346	115.32		MO 08152	98.91
AW 00189	19.97*		CC 13709	50.75		CC 50809	100.83		MO 08153	110.21
AW 00326	39.36		CC 17418	52.77		CC 53409	102.81		MO 08155	89.64
AW 00354	71.86		CC 17446	93.96		CC 53746	85.91		MO 08164	93.18
AW 00496	16.33*		CC 18413	48.48		CC 54609	54.24		MO 08166	94.37
AW 00512	26.92		CC 18509	36.82		CC 55813	110.02		MO 08172	36.97*
AW 00937	19.67*		CC 20209	21.54*		CC 57846	96.64		MO 08173	77.22
BTB 02219	64.59		CC 20309	70.02		CC 64201	82.85		NRB 04027	78.61
BTB 02522	88.37		CC 21101	74.56		CC 64513	75.68		RF 03948	122.21
BTB 03488	36.84		CC 21901	83.90		CC 67423	69.43		RH 00001	92.40
BTB 03690	11.98*		CC 23109	19.07*		CD 02568	51.84		RH 01364	20.38*
BTB 03853	40.89		CC 24201	14.26*		CD 07751	88.29		RH 01808	83.18
BTB 04061	17.41*		CC 24301	25.41		CD 09967	102.68		RH 01882	88.76
BTB 05603	89.62		CC 25501	25.44*		SEW 04290	89.05		RH 01995	61.95
BTB 06663	79.41		CC 25801	20.41*		CD 12030	92.79		RH 02036	61.87
BTB 06837	77.82		CC 26713	52.78		RF 05447	78.10		RH 02136	61.83
BTB 07001	14.76*		CC27201	94.09		DP 01095	36.30*		SB 00671	82.24
BTB 07148	92.25		CC 29209	21.34*		GK 03755	108.28		CC 40996	38.59
BTB 07340	10.27*		CC 29246	35.78		HAN 00271	118.92		SB 01274	81.46
BTB 08555	42.65		CC 29709	75.52		JFD 03933	100.25		SB 01424	39.40
BTB 09194	93.21		CC 30513	43.71		MO 07916	72.66		SB 01670	60.99
BTB 09309	42.60		CC 33809	66.56		KM 00154	55.02		SB 01717	69.03
BTB 09341	65.67		CC 33946	31.28		KM 00452	74.09		SB 01748	71.38
BTB 09397	58.61		CC 34001	90.25		KM 00835	95.26		SB 01855	70.42

MO 08140	65.73		CC 34046	66.09		KM 01136	134.47		SB 01999	96.77
BTB 09912	45.44		CC 36009	101.90		KM 01166	86.56		SB 02020	79.40
BTB 10644	71.05		CC 38113	83.88		KM 01548	28.42*		SB 02033	67.46
BTB 12321	72.59		CC 39001	22.96*		KM 01765	40.56		SB 02112	76.37
BTB 12329	21.55*		CC 40001	59.60		KM 02063	87.50		SB 02117	88.80
BTB 12548	95.28		CC 40109	105.35		KM 02425	64.35		SEW 00597	96.57
BTB 12757	64.76		CC 40146	124.11		KM 03308	65.26		SEW 02215	61.36
BTB 13068	14.01*		CC 40246	32.45*		KM 03728	64.07		SEW 02254	65.79
BTB 13395	24.90*		CC 41401	70.48		KM 05339	97.92		SEW 02362	61.06
BTB 13431	107.27		CC 41709	47.06*		KM 08328	73.52		SEW 02754	90.10
BTB 14321	57.37		CC 42223	38.60*		KM 10090	38.61		SEW 03073	102.15
BTB 15120	80.31		CC 43109	95.50		KM 10848	132.75		SEW 03743	70.37
CC 00075	73.04		CC 43214	54.66		MO 00094	89.85		SEW 03999	64.42
CC 00092	59.30		CC 43309	78.15		MO 00397	33.91		SEW 04032	71.63
CC 00409	43.00		CC 43314	85.95		MO 00719	59.42		SEW 04033	76.36
CC 00509	55.01		CC44201	82.34		MO 00765	84.62		SEW 04036	38.37
CC 00513	40.17		CC 44309	82.76		MO 00936	32.86		SP 00291	43.90
CC 00546	108.62		CC 44546	55.01		MO 00997	31.10*		SPB 01074	21.40*
CC 00801	61.66		CC 45309	111.63		MO 01145	76.94		SPB 02010	66.06
CC 00901	49.37		CC 45522	81.83		MO 01157	62.81		SPB 02157	28.44*
CC 01309	96.94		CC 45609	88.15		MO 01158	51.29		SPB 03583	27.22*
CC 01409	77.19		CC 45613	75.92		MO 01169	122.68		SPB 03996	22.32*
CC 01418	51.43		CC 45801	88.15		MO 01174	94.87		SPB 06632	57.59
CC 01509	102.54		CC 46201	60.75		MO 01175	117.31		SPB 06834	98.24
CC 01513	75.22		CC 46609	77.47		MO 01209	81.84		TL 00276	28.46*
CC 04401	141.62		CC 46646	76.92		MO 01298	77.15		TL 00555	34.52
CC 04601	51.62		CC 46709	56.92		MO 07029	113.86		TL 00757	56.63
CC 04701	90.69		CC 47109	61.14		MO 07058	41.53		TL 00770	23.53*
CC 05601	62.71		CC 47414	75.92		MO 07116	91.54		TL 01004	79.78
CC 06309	72.16		CC 47809	48.21		MO 07123	40.17		TL 01028	36.51
CC 06346	70.68		CC 48009	42.44*		MO 07352	50.66		XBX 00309	49.78
CC 09009	17.89*		CC 48401	70.29		MO 07617	69.00		AC 29603	64.28
CC 10501	41.28		CC 48446	79.86		MO 07680	68.38		SEW 05612	24.67*
CC 11501	48.86		CC 48746	37.19*		MO 07692	92.88		KM 07581	115.31
CC 12046	27.89*		CC 48909	73.18		MO 07745	56.61		CC 41546	98.24
CC 12413	53.63		CC 49701	76.73		MO 08136	41.02		MO 08160	88.72

Table S2. Binding affinities of 38 Maybridge "Rule of Three" fragments at the adenosine A_3 receptor. Values obtained in a fluorescent adenosine receptor antagonist binding assay using whole, live cells expressing the adenosine A_3 receptor. Values represent mean ± s.e.m from 3 separate experiments.

**Ranking**	**Fragment code**	**pK_i**		**Ranking**	**Fragment code**	**pK_i**
1	DP 01095	6.44 ± 0.31		20	CC 23109	4.86 ± 0.38
2	MO 00997	6.10 ± 0.31		21	SPB 03583	4.86 ± 0.50
3	TL 00770	6.00 ± 0.17		22	SPB 03996	4.84 ± 0.50
4	AC 34875	5.92 ± 0.45		23	BTB 12329	4.80 ± 0.24
5	BTB 07340	5.53 ± 0.13		24	SPB 02157	4.78 ± 0.34
6	AC 30454	5.50 ± 0.35		25	CC 09009	4.67 ± 0.30
7	CC 20209	5.47 ± 0.09		26	MO 08172	4.65 ± 0.44
8	CC 39001	5.43 ± 0.05		27	CC 40246	4.61 ± 0.18
9	CC 25501	5.26 ± 0.13		28	BTB 04061	4.49 ± 0.49
10	AW 00937	5.25 ± 0.11		29	CC 29209	4.47 ± 0.50
11	BTB 13068	5.23 ± 0.16		30	BTB 07001	4.40 ± 0.21
12	TL 00276	5.23 ± 0.24		31	SPB 01074	4.38 ± 0.20
13	CC 48746	5.23 ± 0.98		32	RH 01364	4.32 ± 0.20
14	KM 01548	5.19 ± 0.72		33	AW 00189	4.21 ± 0.36
15	CC 24201	5.09 ± 0.37		34	AW 00496	3.97 ± 0.87
16	SEW 05612	5.08 ± 0.20		35	CC 12046	>3
17	BTB 13395	5.02 ± 0.28		36	CC 25801	>3
18	CC 48009	4.99 ± 0.20		37	CC 41709	>3
19	BTB 03690	4.96 ± 0.29		38	CC 42223	>3

Table S3. Binding affinities of DP01095 derivates at the adenosine A_3 and A_1 receptors. Values obtained in a fluorescent adenosine receptor antagonist binding assay using whole, live cells expressing the adenosine A_1 or A_3 receptor. % inhibition values are quoted where less than 50% inhibition of total binding was observed at the top concentration of compound tested. Values represent mean ± s.e.m from *n* separate experiments.

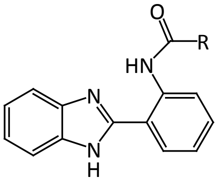

10 - 16

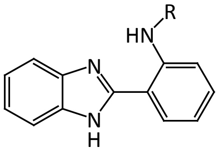

17 - 23

		A_3			A_1		
Compound	R	pK_i	% inhibition	n	pK_i	% inhibition	n
10	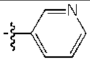	7.01 ± 0.10		4		38.9 ± 8.3	4
11	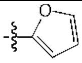	6.78 ± 0.19		4		40.6 ± 7.3	4
12	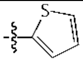	6.59 ± 0.23		4		47.9 ± 9.7	4
13	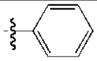	6.27 ± 0.17		5		28.8 ± 18.3	4
14	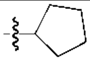		48.2 ± 2.2	4			
15	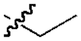		36.8 ± 2.3	4			
16	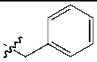		43.0 ± 4.5	4			
17	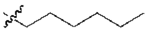	6.27 ± 0.05		5		34.2 ± 5.3	4

18	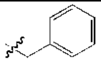		39.0 ± 9.5	5		14.5 ± 5.5	4
19	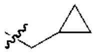		24.0 ± 4.1	4			
20	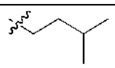		39.0 ± 4.9	4			
21	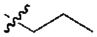		34.2 ± 5.9	4			
22	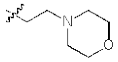		4.9 ± 4.7	4			
23	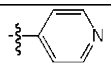		6.5 ± 3.8	4			

GENERAL CHEMISTRY METHODS

Chemicals and solvents of an analytical grade were purchased from commercial suppliers and used without further purification. Reactions were monitored and analyzed by thin layer chromatography on pre-coated aluminium-backed plates (Merck Kieselgel 60 F254) with visualization using UV light (254 and 366 nm) or staining with $KMnO_4$ dip. Flash column chromatography was performed using Merck Kieselgel 60, 230-400 mesh (Merck KgaA) on a Biotage Flashmaster II system. Microwave reactions were carried out in a CEM Discover system fitted with an Explorer 48 autosampler. A dynamic program was used to control the microwave conditions, in which the maximum temperature, pressure, and/or power limit could be specified. Melting points (mp) were recorded on a Mettler Toledo MP50 melting point system and are uncorrected. ^{1}H NMR spectra were recorded on a Bruker-AV 400 at 400.13 MHz or a Bruker AV(II) 500 at 503.13 MHz. ^{13}C NMR spectra were recorded on a Bruker AV(II) 500 with a dual (CH) cryoprobe at 125.8 MHz. Solvents used for NMR analysis (reference peaks listed) were DMSO-d_6 ($(C\underline{H}D_2)_2SO$ at δ_H 2.50 ppm, $(\underline{C}D_3)_2SO$ at 39.52 ppm) and $CDCl_3$ ($C\underline{H}Cl_3$ at δ_H 7.26 ppm, $\underline{C}DCl_3$ at 77.16). Chemical shifts (δ) are recorded in parts per million (ppm). The peaks are described as a singlet (s), doublet (d), doublet of doublets (dd), triplet (t), quadruplet (q), broad (br), multiplet (m), and coupling constants (*J*) are recorded in Hz. High resolution mass spectrometry (HRMS) – time of flight, electrospray (TOF ES +/-) were recorded on a Waters 2795 separation module/micromass LCT platform. RP-HPLC was performed using a Waters 2767 sample manager, Waters 2525 binary gradient module, and visualized at 254 nm and 366 nm with a Waters 2487 dual wavelength absorbance detector. Spectra were analyzed using MassLynx. Analytical reversed-phase high-performance liquid chromatography (RP-HPLC) was used to determine that the purity of compounds tested in biological systems was≥ 95% (the retention times (R_t) of these compounds are reported). Analytical RP-HPLC was performed using a YMC-Pack C8 column (150 mm × 4.6 mm × 5μ m) at a flow rate of 1 mL/min, and using a method of 0 min 10%B, 1 min 10%B, linear gradient to 20 min 95%B, 21 min 95%B, followed by column re-equilibration to 10%B in solvent A (solvent A = 0.05% trifluoroacetic acid in

H_2O, solvent B = 0.05% trifluoroacetic acid in 9:1 *v*:*v* CH_3CN:H_2O).

Synthesis and characterization of compounds 1 – 7

3-Aminoquinoline (**5**) and 1-(4-methoxyphenyl)-1*H*-imidazole (**6**) were purchased from Sigma Aldrich.

N-[2-(4-Methoxyphenyl)-1-oxo-1*H*,2*H*-[1,2,4]triazolo[4,3-*a*]quinoxalin-4-yl]benzamide **1**

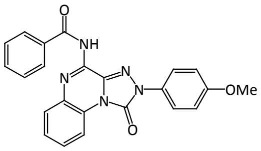

Synthesized according to literature.[3] mp: 202 - 204 °C (lit. 203–205 °C).[3] ^{1}H NMR (400 MHz, $CDCl_3$) δ 3.86 (s, 3H), 7.02 (d, *J* = 9.3 Hz, 2H), 7.50 – 7.65 (m, 5H), 7.93–7.96 (m, 3H), 8.01 (d, *J* = 7.5 Hz, 2H), 8.84 (d, *J* = 7.5 Hz, 1H), 8.93 (s, 1H). HRMS (*m*/*z*): $[M+H]^+$ calcd. for $C_{23}H_{18}N_5O_3$, 412.1404; found, 412.1393. Analytical HPLC R_t = 17.77 min.

2-(4-Methoxyphenyl)-5*H*-[1,2,4]triazolo[4,3-*a*]quinoxaline-1,4-dione **2**

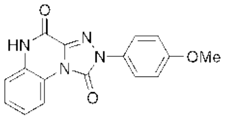

Synthesized according to literature.[4] mp: > 300 °C (lit. > 300 °C).[4] ^{1}H NMR (400 MHz, DMSO-d_6) δ 3.81 (s, 3H), 7.11 (d, *J* = 9.1 Hz, 2H), 7.25-7.37 (m, 3H), 7.87 (d, *J* = 9.1 Hz, 2H), 8.60 (d, *J* = 7.9 Hz, 1H), 11.94 (s, 1H). HRMS (*m*/*z*): $[M+H]^+$ calcd. for $C_{16}H_{13}N_4O_3$, 309.0982; found, 309.0995. Analytical HPLC R_t = 14.47 min.

(*Z*)-3-[2-(4-Methoxyphenyl)hydrazin-1-ylidene]-1,4-dihydroquinoxalin-2-one **3**

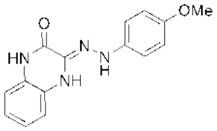

Synthesized according to literature.[4] mp: 230–231 °C (lit. 231–233 °C).[4] ^{1}H NMR (400 MHz, DMSO-d_6) δ 3.65 (s, 3H, tautomer A), 3.70 (s, 3H, tautomer B), 6.76–7.26 (m, 16H, tautomer A and B), 7.44 (d, *J* = 3.5 Hz, tautomer B, 1H), 8.48 (s, 1H, tautomer A), 9.32 (d, *J* = 3.5 Hz, tautomer B, 1H), 9.45 (s, 1H, tautomer A), 11.00 (s, 1H, tautomer A), 12.22 (s, 1H, tautomer B). HRMS (*m*/*z*): $[M+H]^+$ calcd. for $C_{15}H_{15}N_4O_2$, 283.1190; found 238.1198. Analytical HPLC R_t = 13.15 min.

N-(Quinolin-3-yl)benzamide **4**

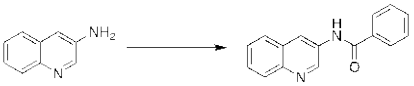

To a solution of 3-aminoquinoline (0.35 mmol) in *N*,*N*-dimethylformamide (DMF) (2 mL) was added a solution of benzoic acid (0.48 mmol), 2-(7-aza-1*H*-benzotriazole-1-yl)-1,1,3,3-tetramethyluronium hexafluorophosphate (HATU) (0.48 mmol), and *N*,*N*-diisopropylethylamine (DIEA) (1.9 mmol) in DMF (1 mL). The solution was stirred for 6 h at rt, then ethyl acetate and water were added. The organic layer was separated and washed with saturated aqueous sodium bicarbonate, saturated brine, and water, dried over magnesium sulfate, and evaporated to give the crude product. Recrystalization gave the pure product **4** (56 mg, 65%) as pale yellow crystals. mp: 199–200 °C (lit. 198–199 °C).[5] ^{1}H NMR (400 MHz, DMSO-d_6) δ 7.54–7.67 (m, 5H), 7.95 (d, *J* = 8.2 Hz, 1H), 8.01 (d, *J* = 8.4 Hz, 1H), 8.08 (m, 2H), 8.90 (d, *J* = 2.4 Hz, 1H), 9.22 (d, *J* = 2.5 Hz, 1H), 10.74 (s, 1H); ^{13}C NMR (DMSO-d_6) δ 123.37, 126.99, 127.80, 127.82, 127.97, 128.49, 128.62, 131.92, 133.00, 134.39, 144.45, 145.54, 166.22. HRMS (*m*/*z*): $[M+H]^+$ calcd. for $C_{16}H_{13}N_2O$, 249.1028; found, 249.1035. Analytical RP-HPLC R_t =11.14 min.

N-(4-Methoxyphenyl)acetamide **7**

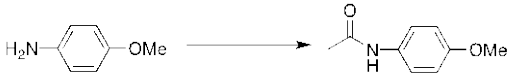

4-Methoxyaniline (1 mmol) was dissolved in ethyl acetate (5 mL) and DIEA (7 mmol), followed by addition of acetic anhydride (5 mmol). The reaction was stirred at room temperature overnight. The solvent was removed under reduced pressure and the residue dissolved in ethyl acetate. The organics were washed three times with water, dried over magnesium sulfate, and the solvent evaporated under reduced pressure. The product was crystalized from petroleum ether/ethyl acetate to give **7** (53 mg, 32%) as pale purple crystals. mp: 127–128 ^{o}C (lit. 126–127 ^{o}C).[6] ^{1}H NMR (400 MHz, DMSO-d_6) δ 1.99 (s, 3H), 3.71 (s, 3H), 6.85 (d, *J* = 9.1 Hz, 2H), 7.47 (d, *J* = 9.1 Hz, 2H), 9.76 (s, 1H). HRMS (*m*/*z*): $[M+H]^+$ calcd. for $C_9H_{11}NO_2$, 166.0868; found, 166.0858. Analytical RP-HPLC R_t = 10.14 min.

Synthesis and characterization of compounds DP 01095, 8, and 9

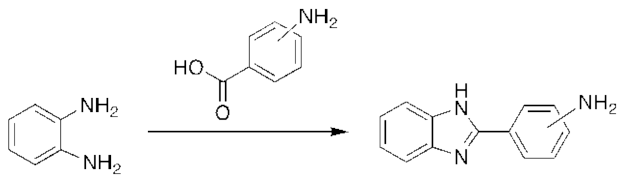

General Procedure A: *o*-Phenylenediamine (0.6 mmol) and the amino benzoic acid (0.65 mmol) were combined with polyphosphoric acid (2 g), and the resulting paste heated at 180 ^{o}C for 18 h. The mixture was cooled to 80-90 ^{o}C, poured into cold water, and adjusted to pH 9-10 by addition of 2M NaOH. The aqueous solution was extracted three times with ethyl acetate, the organic layers combined, dried over magnesium sulfate, and evaporated to give the crude reaction product. This was purified by flash silica column chromatography eluting with 0 to 10% methanol in dichloromethane with 1% triethylamine. After evaporation of the solvent from the desired column fractions, 3M HCl in methanol was added, and the product was recrystalized as the hydrochloride salt.

2-(1*H*-Benzimidazol-2-yl)aniline hydrochloride DP 01095

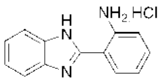

o-Phenylenediamine (30 mmol), 2-amino benzoic acid (30 mmol), and polyphosphoric acid (100 g) gave DP01095 (3.449 g, 55%) as a pale brown solid according to a scaled-up General Procedure A. mp: decomposed > 280 ^{o}C. ^{1}H NMR (500 MHz, DMSO-d_6) δ 6.66 (t, *J* = 8.1 Hz, 1H), 6.85 (dd, *J* = 1.0, 8.3 Hz, 1H), 7.14–7.23 (m, 3H), 7.59 (br m, 2H), 7.86 (dd, *J* = 1.4, 7.9 Hz, 1H), 12.70 (br s, 1H). ^{13}C NMR (DMSO-d_6) δ 110.05, 110.92, 114.99, 116.13, 118.18, 121.51, 122.31, 127.28, 130.43, 133.55, 143.02, 148.27, 152.57. HRMS (*m*/*z*): [M+H]$^+$ calcd. for $C_{13}H_{12}N_3$, 210.1026; found, 210.1016. Analytical RP-

HPLC R_t = 8.94 min.

3-(1*H*-Benzimidazol-2-yl)aniline hydrochloride **8**.

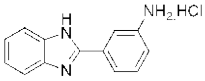

o-Phenylenediamine and 3-amino benzoic acid gave **8** (47 mg, 37%) as green crystals according to General Procedure A. mp: decomposed > 295 ^oC. ^1H NMR (500 MHz, DMSO-d_6) δ 7.52 (br m, 1H), 7.57 (m, 2H), 7.69 (t, J = 7.9 Hz, 1H), 7.86 (m, 2H), 8.02 (br s, 1H), 8.17 (br m, 1H). ^{13}C NMR (DMSO-d_6) δ 114.15, 119.32, 123.97, 124.51, 125.09, 126.07, 130.91, 132.01, 138.63, 148.11. HRMS (*m*/*z*): $[M+H]^+$ calcd. for $C_{13}H_{12}N_3$, 210.1026; found, 210.1030. Analytical RP-HPLC R_t = 7.68 min.

4-(1*H*-Benzimidazol-2-yl)aniline hydrochloride **9**.

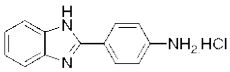

o-Phenylenediamine and 4-amino benzoic acid gave **9** (94 mg, 75%) as a yellow solid according to General Procedure A. mp: decomposed > 295 ^oC. ^1H NMR (500 MHz, DMSO-d_6) δ 6.89 (d, J = 8.8 Hz, 2H), 7.47 (m, 2H), 7.73 (m, 2H), 8.19 (d, J = 8.9 Hz, 2H). ^{13}C NMR (DMSO-d_6) δ 109.78, 113.22, 114.98, 125.25, 130.02, 131.47, 149.38, 151.95. HRMS (*m*/*z*): $[M+H]^+$ calcd. for $C_{13}H_{12}N_3$, 210.1026; found, 210.1023. Analytical RP-HPLC R_t = 8.92 min.

Synthesis and characterization of compounds 10 – 16

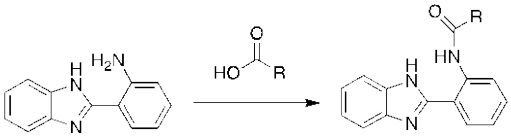

General Procedure B. To a solution of 2-(1*H*-benzimidazol-2-yl)aniline DP 01095 (0.24 mmol) in DMF (0.5 mL) was added a solution of the carboxylic acid (0.48 mmol), HATU (0.48 mmol), and DIEA (1.9 mmol) in DMF (1 mL). The solution was stirred for 6 h at rt, evaporated under reduced pressure to remove most of the DMF, and then ethyl acetate and water were added. The organic layer was separated and washed with saturated aqueous sodium bicarbonate, saturated brine, and water, dried over magnesium sulfate, and evaporated to give the crude product. This residue was purified by flash silica column chromatography eluting with 0-10% methanol in dichloromethane, and then recrystalized to give the desired product.

N-(2-(1*H*-Benzimidazol-2-yl)phenyl)nicotinamide **10**

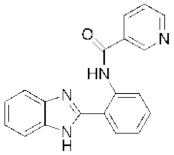

Nicotinic acid gave **10** (19 mg, 25%) as a white solid according to General Procedure B. mp: 261 ^{o}C.; ^{1}H NMR (500 MHz, DMSO-d_6) δ 7.34 (m, 3H), 7.57 (t, *J* = 7.9 Hz, 1H), 7.64 (br m, 1H), 7.76 (m, 2H), 8.21 (dd, *J* = 1.4, 8.0 Hz, 1H), 8.56 (dt, *J* = 1.7, 2.3, 8.0 Hz, 1H), 8.86 (dd, *J* = 1.6, 4.8 Hz, 1H), 8.90 (dd, *J* = 1.0, 8.4 Hz, 1H), 9.39 (dd, *J* = 0.7, 2.3 Hz, 1H), 13.33 (br s, 1H), 14.21 (s, 1H). ^{13}C NMR (DMSO-d_6) δ 111.79, 115.79, 118.22, 120.05, 122.64, 123.55, 123.76, 124.08, 127.36, 130.45, 130.97, 133.39, 135.04, 138.17, 141.79, 148.69, 150.93, 152.65, 163.51. HRMS (*m*/*z*): $[M+H]^+$ calcd. for $C_{19}H_{15}N_4O$, 315.1240; found, 315.1260. Analytical RP-HPLC R_t = 10.12 min.

N-(2-(1*H*-Benzimidazol-2-yl)phenyl)furan-2-carboxamide **11**

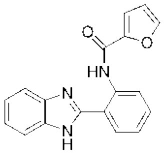

2-Furoic acid gave **11** (17 mg, 23%) as a white solid according to General Procedure B. mp: 263–265 °C. ^{1}H NMR (500 MHz, DMSO-d_6) δ 6.80 (dd, *J* = 1.7, 3. Hz, 1H), 7.32 (m, 3H), 7.39 (dd, *J* = 0.7, 3.5 Hz, 1H), 7.54 (m, 1H), 7.62 (m, 1H), 7.89 (d, *J* = 7.2 Hz, 1H), 8.18 (m, 1H), 8.18 (dd, *J* = 1.4, 8.0 Hz, 1H), 8.84 (dd, *J* = 1.0, 8.4 Hz, 1H), 13.25 (br s, 1H), 14.18 (s, 1H). ^{13}C NMR (DMSO-d_6) δ 111.28, 112.34, 114.69, 115.29, 118.37, 119.72, 122.06, 122.91, 123.30, 127.01, 130.51, 133.18, 137.73, 141.68, 146.01, 147.69, 150.50, 155.93. HRMS (*m*/*z*): $[M+H]^+$ calcd. for $C_{18}H_{14}N_3O_2$, 304.1081; found, 304.1068. Analytical RP-HPLC R_t = 12.09 min.

N-(2-(1*H*-Benzimidazol-2-yl)phenyl)thiophene-2-carboxamide **12**

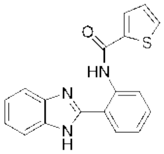

Thiophene-2-carboxylic acid gave **12** (15 mg, 20%) as a white solid according to General Procedure B. mp: 261 °C. ^{1}H NMR (500 MHz, DMSO-d_6) δ 7.30–7.35 (m, 3H), 7.41 (m, 1H), 7.54 (m, 1H), 7.65 (br m, 1H), 7.87 (br m, 1H), 7.97 (dd, *J* = 1.2, 5.0 Hz, 1H), 8.16 (dd, *J* = 1.1, 3.8 Hz, 1H), 8.20 (dd, *J* = 1.4, 8.0 Hz, 1H), 8.81 (dd, *J* = 1.1, 8.4 Hz, 1H), 13.31 (br s, 1H), 14.14 (s, 1H). ^{13}C NMR (DMSO-d_6) δ 111.74, 115.37, 118.37, 119.81, 122.52, 123.19, 123.65, 127.35, 128.68, 128.92, 130.95, 132.36, 133.46, 138.23, 140.44, 141.84, 150.99, 159.87. HRMS (*m*/*z*): $[M+H]^+$ calcd. for $C_{18}H_{14}N_3OS$, 320.0852; found, 320.0880. Analytical RP-HPLC R_t = 13.85 min.

N-(2-(1*H*-Benzimidazol-2-yl)phenyl)benzamide **13**

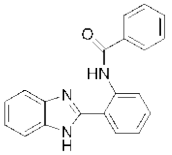

Benzoic acid gave **13** (19 mg, 25%) as a white solid according to General Procedure B. mp: 243-244 ^{o}C.; ^{1}H NMR (500 MHz, DMSO-d_6) δ 7.33 (m, 3H), 7.57 (t, *J* = 8.6 Hz, 1H), 7.63 (d, *J* = 7.3 Hz, 1H), 7.69–7.72 (m, 3H), 7.80 (d, *J* = 7.4 Hz, 1H), 8.20 (dd, *J* = 1.3, 7.9 Hz, 1H), 8.26 (m, 2H), 8.95 (dd, *J* = 1.0, 8.4 Hz, 1H), 13.30 (s, 1H), 14.09 (s, 1H). ^{13}C NMR (DMSO-d_6) δ 111.68, 115.67, 118.29, 119.98, 122.53, 123.20, 123.68, 127.36, 127.41, 129.03, 130.92, 132.15, 133.44, 134.86, 138.49, 141.89, 150.99, 164.99. HRMS (*m*/*z*): $[M+H]^+$ calcd. for $C_{20}H_{16}N_3O$, 314.1288; found, 314.1278. Analytical RP-HPLC R_t = 13.90 min.

N-(2-(1*H*-Benzimidazol-2-yl)phenyl)cyclopentanecarboxamide **14**

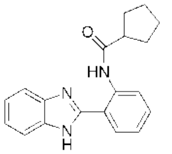

Cyclopentane carboxylic acid gave **14** (17 mg, 23%) as a white solid according to General Procedure B. mp: 208–209 ^{o}C. ^{1}H NMR (500 MHz, DMSO-d_6) δ 1.65 (m, 2H), 1.74 (m, 2H), 1.89 (m, 2H), 2.02 (m, 2H), 2.95 (m, 1H), 7.23 (t, *J* = 8.3 Hz, 1H), 7.29 (br m, 2H), 7.47 (t, *J* = 8.5 Hz, 1H), 7.65 (br m, 2H), 8.11 (dd, *J* = 1.4, 7.9 Hz, 1H), 8.71 (dd, *J* = 1.0, 8.4 Hz, 1H), 13.07 (s, 1H), 13.17 (br s, 1H). ^{13}C NMR (DMSO-d_6) δ 25.62, 29.86, 47.20, 111.54, 115.22, 118.48, 119.89, 122.27, 122.61, 123.38, 127.27, 130.67, 133.48, 138.56, 142.07, 150.92, 174.54. HRMS (*m*/*z*): $[M+H]^+$ calcd. for $C_{19}H_{20}N_3O$, 306.1601; found 306.1594. Analytical RP-HPLC R_t = 13.14 min.

N-(2-(1*H*-Benzimidazol-2-yl)phenyl)propionamide **15**

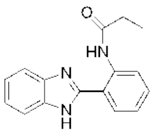

Propanoic acid gave **15** (11 mg, 17%) as a white solid according to General Procedure B. mp: 226–227 °C. ^{1}H NMR (500 MHz, DMSO-d_6) δ 1.25 (t, *J* = 7.6 Hz, 3H), 2.53 (q, *J* = 7.6 Hz, 2H), 7.25 (m, 1H), 7.29 (m, 2H), 7.48 (t, *J* = 8.6 Hz, 1H), 7.66 (br m, 2H), 8.11 (dd, *J* = 1.4, 7.9 Hz, 1H), 8.71 (dd, *J* = 1.1, 8.4 Hz, 1H), 13.03 (s, 1H), 13.17 (br s, 1H). ^{13}C NMR (DMSO-d_6) δ 9.58, 31.10, 111.70, 115.22, 118.48, 119.85, 122.68, 123.20, 123.30, 127.30, 130.68, 133.61, 138.42, 142.28, 150.89, 172.18. HRMS (*m*/*z*): $[M+H]^+$ calcd. for $C_{16}H_{16}N_3O$, 266.1288; found, 266.1306. Analytical RP-HPLC R_t = 14.42 min.

N-(2-(1*H*-Benzimidazol-2-yl)phenyl)-2-phenylacetamide **16**

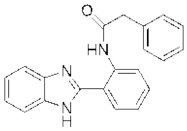

Phenylacetic acid gave **16** (35 mg, 45%) as a white solid according to General Procedure B. mp: 245 °C. ^{1}H NMR (500 MHz, DMSO-d_6) δ 3.85 (s, 2H), 7.24 (m, 2H), 7.30 (dd, *J* = 3.2, 6.0 Hz, 2H), 7.33 (t, *J* = 7.6 Hz, 2H), 7.44–7.48 (m, 3H), 7.68 (br m, 2H), 8.10 (dd, *J* = 1.4, 8.0 Hz, 1H), 8.68 (dd, *J* = 1.1, 8.5 Hz, 1H), 13.17 (br s, 2H). ^{13}C NMR (DMSO-d_6) δ 45.44, 111.03, 115.47, 117.52, 119.88, 122.83, 122.92, 126.89, 127.30, 128.57, 129.43, 130.63, 135.21, 135.44, 138.30, 142.29, 150.77, 169.67. HRMS (*m*/*z*): $[M+H]^+$ calcd. for $C_{21}H_{18}N_3O$, 328.1444; found, 328.1444. Analytical RP-HPLC R_t = 11.85 min.

Synthesis and characterization of compounds 17 – 23

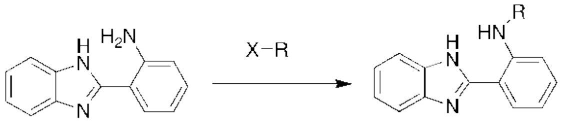

General Procedure C. A solution of 2-(1*H*-benzimidazol-2-yl)aniline DP 01095 (1 mmol) and the bromo- or chloro- substituted compound (0.4 mmol) in DMF (1 mL) was reacted in the microwave for 5 min using a dynamic control program with the temperature held at 150 ^{o}C, maximum power set at 300 W, and maximum pressure set at 150 psi. The solvent was evaporated and the residue dissolved in ethyl acetate, washed with water, saturated brine, and then water. The organic layer was dried over magnesium sulfate, and evaporated to give the crude product. This residue was purified by flash silica column chromatography eluting with 0–60% ethyl acetate in petroleum ether, and then recrytalized to give the desired product.

2-(1*H*-Benzimidazol-2-yl)-*N*-hexylaniline **17**

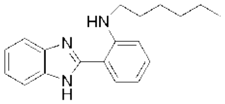

6-Bromohexane gave **17** (41 mg, 35%) as a white solid according to General Procedure C. mp: 111−112 ^{o}C. ^{1}H NMR (500 MHz, $CDCl_3$) δ 0.92 (t, *J* = 7.0 Hz, 3H), 1.37 (m, 4H), 1.52 (m, 2H), 1.80 (m, 2H), 3.29 (t, *J* = 7.1 Hz, 2H), 6.68 (t, *J* = 7.5 Hz, 1H), 6.82 (d, *J* = 8.6 Hz, 1H), 7.24–7.27 (m, 2H), 7.29 (m, 1H), 7.56 (dd, *J* = 1.2, 7.8 Hz, 1H), 7.60 (br m, 2H), 9.23 (br s, 1H). ^{13}C NMR ($CDCl_3$) δ 14.08, 22.64, 26.97, 29.04, 31.63, 43.49, 110.62, 111.82, 114.93, 122.61, 126.54, 131.27, 147.92, 152.01. HRMS (*m*/*z*): $[M+H]^+$ calcd. for $C_{29}H_{24}N_3$, 294.1965; found, 294.1951. Analytical RP-HPLC R_t = 16.10 min.

2-(1*H*-Benzimidazol-2-yl)-*N*-benzylaniline **18**

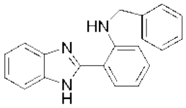

Benzyl bromide gave **18** (48 mg, 40%) as a white solid according to a modified General Procedure C (reaction was heated for 2 min at 90 ^{o}C instead of 5 min at 150 ^{o}C, as reaction at 150 ^{o}C for 5 min resulted in a di-alkylated product). mp: 158–160 ^{o}C. ^{1}H NMR (500 MHz, $CDCl_3$) δ 4.62 (s, 2H), 6.68–6.73 (m, 2H), 7.22 (m, 1H), 7.25–7.27 (m, 3H), 7.34 (m, 2H), 7.43 (m, 2H), 7.59 (dd, *J* = 1.1, 7.6 Hz, 1H), 7.61 (br m, 2H), 9.45 (br s, 1H). ^{13}C NMR ($CDCl_3$) δ 47.04, 110.81, 112.03, 115.18, 122.66, 126.49, 126.81, 126.86, 128.52, 131.25, 139.51, 148.01, 152.09. HRMS (*m*/*z*): $[M+H]^+$ calcd. for $C_{20}H_{18}N_3$, 300.1495; found, 300.1496. Analytical RP-HPLC R_t = 14.34 min.

2-(1*H*-Benzimidazol-2-yl)-*N*-(cyclopropylmethyl)aniline **19**

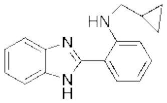

1-(Bromomethyl)cyclopropane gave **19** (32 mg, 30%) as an off white solid according to General Procedure C. mp: 153 ^{o}C. ^{1}H NMR (500 MHz, $CDCl_3$) δ 0.34 (m, 2H), 0.60 (m, 2H), 1.24 (m, 1H), 3.17 (d, *J* = 6.8 Hz, 2H), 6.68 (t, *J* = 7.5 Hz, 1H), 6.79 (d, *J* = 8.3 Hz, 1H), 7.24–7.26 (m, 2H), 7.29 (m, 1H), 7.54 (dd, *J* = 1.5, 7.8 Hz, 1H), 7.60 (br m, 2H), 9.15 (br s, 1H). ^{13}C NMR ($CDCl_3$) δ 3.63, 10.67, 48.00, 110.63, 111.55, 114.80, 122.54, 126.70, 131.25, 148.13, 152.16. HRMS (*m*/*z*): $[M+H]^+$ calcd. for $C_{17}H_{18}N_3$, 264.1495; found, 264.1505. Analytical RP-HPLC R_t = 12.92 min.

2-(1*H*-Benzimidazol-2-yl)-*N*-isopentylaniline **20**

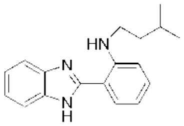

1-Bromo-3-methyl butane gave **20** (38 mg, 34%) as a white solid according to General Procedure C. mp: 144–146 °C. ^{1}H NMR (500 MHz, $CDCl_3$) δ 0.99 (d, *J* = 6.6 Hz, 6H), 1.68 (q, *J* = 7.6 Hz, 2H), 1.84 (m, 1H), 3.30 (t, *J* = 7.3 Hz, 2H), 6.67 (t, *J* = 7.5 Hz, 1H), 6.79 (d, *J* = 8.5 Hz, 1H), 7.23 – 7.28 (m, 2H), 7.30 (m, 1H), 7.53 (dd, *J* = 1.5, 7.8 Hz, 1H), 7.59 (br m, 2H), 9.10 (br s, 1H). ^{13}C NMR ($CDCl_3$) δ 22.64, 26.14, 38.06, 41.40, 110.40, 111.34, 114.52, 122.54, 126.59, 131.29, 148.23, 152.19. HRMS (*m*/*z*): $[M+H]^+$ calcd. for $C_{18}H_{22}N_3$, 280.1808; found, 280.1819. Analytical RP-HPLC R_t = 13.14 min.

2-(1*H*-Benzimidazol-2-yl)-*N*-propylaniline **21**

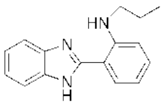

1-Bromopropane gave **21** (33 mg, 33%) as a white solid according to General Procedure C. mp: 136–137 °C. ^{1}H NMR (500 MHz, $CDCl_3$) δ 1.09 (t, *J* = 7.4 Hz, 3H), 1.80 (m, 2H), 3.27 (t, *J* = 7.1 Hz, 2H), 6.67 (t, *J* = 7.5 Hz, 1H), 6.80 (d, *J* = 8.4 Hz, 1H), 7.23 – 7.27 (m, 2H), 7.29 (m, 1H), 7.53 (dd, *J* = 1.5, 7.8 Hz, 1H), 7.60 (br m, 2H), 9.11 (br s, 1H). ^{13}C NMR ($CDCl_3$) δ 11.85, 22.41, 45.02, 110.43, 111.47, 114.60, 122.56, 126.56, 131.28, 148.20, 152.16. HRMS (*m*/*z*): $[M+H]^+$ calcd. for $C_{16}H_{18}N_3$, 252.1495; found, 252.1499. Analytical RP-HPLC R_t = 14.42 min.

2-(1*H*-Benzimidazol-2-yl)-*N*-(2-morpholinoethyl)aniline **22**

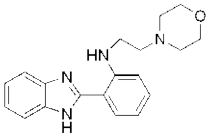

N-(2-Chloroethyl)morpholine hydrochloride gave **22** (37 mg, 29%) as a pale yellow solid according to a modified General Procedure C (0.5 mmol of DIEA was added to the reaction mixture to neutralize the hydrochloride salt starting material) (purified by flash silica column chromatography eluting with 0-10% methanol in dichloromethane). mp: 109–111 °C.; ^{1}H NMR (500 MHz, $CDCl_3$) δ 2.62 (m, 4H), 2.79 (t, *J* = 6.5 Hz, 2H), 3.44 (t, *J* = 6.5 Hz, 2H), 3.81 (t, *J* = 9.3 Hz, 4H), 6.70 (t, *J* = 7.4 Hz, 1H), 6.80 (d, *J*

= 8.3 Hz, 1H), 7.23 – 7.27 (m, 2H), 7.31 (m, 1H), 7.56 (dd, *J* = 1.5, 7.8 Hz, 1H), 7.60 (br m, 2H), 8.94 (br s, 1H). ^{13}C NMR ($CDCl_3$) δ 40.10, 40.94, 53.52, 57.07, 67.04, 111.13, 111.52, 115.04, 122.60, 126.80, 131.29, 147.89, 152.00. HRMS (*m*/*z*): $[M+H]^+$ calcd. for $C_{19}H_{23}N_4O$, 323.1866; found, 323.1887. Analytical RP-HPLC R_t = 8.02 min.

N-(2-(1*H*-Benzimidazol-2-yl)phenyl)pyridin-4-amine **23**

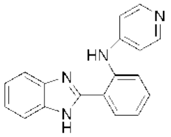

4-Chloropyridine hydrochloride gave **23** (6 mg, 5%) as a pale yellow solid according to a modified General Procedure C (0.5 mmol of DIEA was added to the reaction mixture to neutralize the hydrochloride salt starting material) (purified by flash silica column chromatography eluting with 0 – 10% methanol in dichloromethane). mp: 275–277 ^{o}C. ^{1}H NMR (500 MHz, DMSO-d_6) δ 7.16–7.20 (m, 3H), 7.26 (m, 2H), 7.47 (t, *J* = 7.7 Hz, 1H), 7.57 (d, *J* = 7.7 Hz, 1H), 7.67 (dd, *J* = 0.9, 8.3 Hz, 1H), 7.76 (d, *J* = 7.6 Hz, 1H), 8.10 (dd, *J* = 1.4, 7.9 Hz, 1H), 8.33 (d, *J* = 5.6 Hz, 2H), 11.50 (s, 1H), 13.08 (s, 1H). ^{13}C NMR (DMSO-d_6) δ 111.43, 116.43, 118.23, 118.63, 121.31, 122.07, 123.24, 128.50, 130.62, 133.55, 140.09, 142.33, 148.77, 150.35, 151.02. HRMS (*m*/*z*): $[M+H]^+$ calcd. for $C_{18}H_{15}N_4$, 287.1291; found, 287.1295. Analytical RP-HPLC R_t = 7.20 min.

SUPPLEMENTAL REFERENCES

References not listed here can be found in the reference list in the main text.

Baraldia, P. G., Cacciaria, B., Romagnolia, R., Varanib, K., Merighib, S., Gessib, S., Boreab, P. A., Leungc, E., Hickeyd, S. L., Spallutoe, G. (2000) Synthesis and preliminary biological evaluation of [^{3}H]-MRE-3008-F20: the first high affinity radioligand antagonist for the human A_3 adenosine receptors, *Bioorg. Med. Chem. Lett.* 10, 209-211.

Christopoulos, A., Parsons, A. M., Lew, M. J., El-Fakahany, E. E. (1999) The assessment of antagonist potency under conditions of transient response kinetics, *Eur. J. Pharmacol. 382*, 217-27.

Cole, A. G., Stauffer, T. M., Rokosz, L. L., Metzger, A., Dillard, L. W., Zeng, W., Henderson, I. (2009) Synthesis of 2-amino-5-benzoyl-4-(2-furyl)thiazoles as adenosine A_{2A} receptor antagonists, *Bioorg. Med. Chem. Lett. 19*, 378-381.

Jung, K.Y., Kim, S. K., Gao, Z. G., Gross, A. S., Melman, N., Jacobson, K. A., Kim, Y. C. (2004) Structure-activity relationships of thiazole and thiadiazole derivatives as potent and selective human adenosine A_3 receptor antagonists, *Bioorg. Med. Chem. 12*, 613-623.

Lenzi, O., Colotta, V., Catarzi, D., Varano, F., Filacchioni, G., Martini, C., Trincavelli, L., Ciampi, O., Varani, K., Marighetti, F., Morizzo, E., Moro, S. (2006) 4-amido-2-aryl-1,2,4-triazolo[4,3-*a*]quinoxalin-1-ones as new potent and selective human A_3 adenosine receptor antagonists. Synthesis, pharmacological evaluation, and ligand-receptor modeling studies, *J. Med. Chem. 49*, 3916-3925.

Lenzi, O., Colotta, V., Catarzi, D., Varano, F., Poli, D., Filacchioni, G., Varani, K., Vincenzi, F., Borea, P. A., Paoletta, S., Morizzo, E., Moro, S. (2009) 2-Phenylpyrazolo 4,3-d pyrimidin-7-one as a New Scaffold To Obtain Potent and Selective Human A_3 Adenosine Receptor Antagonists: New Insights into the Receptor-Antagonist Recognition, *J. Med. Chem. 52*, 7640-7652.

Li, A.H., Moro, S., Melman, N., Ji, X.D., Jacobson, K.A. (1998) Structure-activity relationships and molecular modeling of 3,5-diacyl-2,4-dialkylpyridine derivatives as selective A_3 adenosine receptor antagonists, *J. Med. Chem. 41*, 3186-3201.

Okamura, T., Kurogi, Y., Hashimoto, K., Sato, S., Nishikawa, H., Kiryu, K., Nagao, Y. (2004) Structure-activity relationships of adenosine A_3 receptor ligands: new potential therapy for the treatment of glaucoma, *Bioorg. Med. Chem. Lett. 14*, 3775-3779.

Ozola, V., Thorand, M., Diekmann, M., Qurishi, R., Schumacher, B., Jacobson, K. A., Müller, C. E. (2003) 2-Phenylimidazo[2,1-i]purin-5-ones: Structure–Activity relationships and characterization of potent and selective inverse agonists at Human A_3 adenosine receptors, *Bioorg. Med. Chem. 11*, 347-356.

Press, N. J., Keller, T. H., Tranter, P., Beer, D., Jones, K., Faessler, A., Heng, R., Lewis, C., Howe, T., Gedeck, P., Mazzoni, L., Fozard, J. R. (2004) New highly potent and selective adenosine A_3 receptor antagonists, *Curr. Top. Med. Chem. 4*, 863-870.

Saki, M., Tsumuki, H., Nonaka, H., Shimada, J. & Ichimura, M. (2002) KF26777 (2-(4-bromophenyl)-7,8-dihydro-4-propyl-1H-imidazo 2,1-*i*-purin-5(4H)-one dihydrochloride), a new potent and selective adenosine A_3 receptor antagonist, *Eur. J. Pharmacol. 444*, 133-141.

Scheiff, A. B., Yerande, S. G., El-Tayeb, A., Li, W., Inamdar, G. S., Vasu, K. K., Sudarsanam, V., Müller, C. E. (2010) 2-Amino-5-benzoyl-4-phenylthiazoles: Development of potent and selective adenosine A_1 receptor antagonists. *Bioorg. Med. Chem. 18*, 2195-2203.

van Muijlwijk-Koezen, J. E., Timmerman, H., Link, R., van der Goot, H., Ijzermani, A.P. (1998) A novel class of adenosine A_3 receptor ligands. 2. Structure affinity profile of a series of isoquinoline and quinazoline compounds *J. Med. Chem. 41*, 3994-4000.

van Muijlwijk-Koezen, J.E., Timmerman, H., van der Goot, H., Menge, W. M., Frijtag Von Drabbe Künzel, J., de Groote, M., IJzerman, A.P. (2000) Isoquinoline and quinazoline urea analogues as antagonists for the human adenosine A_3 receptor, *J. Med. Chem. 43*, 2227-2238.

van Rhee, A. M., Jiang, J.L., Melman, N., Olah, M. E., Stiles, G. L., Jacobson, K. A. (1996) Interaction of 1,4-dihydropyridine and pyridine derivatives with adenosine receptors: selectivity for A_3 receptors, *J. Med. Chem. 39*, 2980-2989
